# Novel Deep Learning Model for Glaucoma Detection Using Fusion of Fundus and Optical Coherence Tomography Images

**DOI:** 10.3390/s25144337

**Published:** 2025-07-11

**Authors:** Saad Islam, Ravinesh C. Deo, Prabal Datta Barua, Jeffrey Soar, U. Rajendra Acharya

**Affiliations:** 1 Artificial Intelligence Applications Laboratory, School of Mathematics, Physics and Computing, University of Southern Queensland, Springfield, QLD 4300, Australia; rajendra.acharya@unisq.edu.au; 2 School of Business, University of Southern Queensland, Springfield, QLD 4300, Australia; prabal.barua@unisq.edu.au (P.D.B.); jeffrey.soar@unisq.edu.au (J.S.); 3 Faculty of Engineering and Information Technology, University of Technology Sydney, Sydney, NSW 2007, Australia

**Keywords:** glaucoma, fundus, OCT, deep learning, artificial intelligence, fusion, automated detection

## Abstract

Glaucoma is a leading cause of irreversible blindness worldwide, yet early detection can prevent vision loss. This paper proposes a novel deep learning approach that combines two ophthalmic imaging modalities, fundus photographs and optical coherence tomography scans, as paired images from the same eye of each patient for automated glaucoma detection. We develop separate convolutional neural network models for fundus and optical coherence tomography images and a fusion model that integrates features from both modalities for each eye. The models are trained and evaluated on a private clinical dataset (Bangladesh Eye Hospital and Institute Ltd.) consisting of 216 healthy eye images (108 fundus, 108 optical coherence tomography) from 108 patients and 200 glaucomatous eye images (100 fundus, 100 optical coherence tomography) from 100 patients. Our methodology includes image preprocessing pipelines for each modality, custom convolutional neural network/ResNet-based architectures for single-modality analysis, and a two-branch fusion network combining fundus and optical coherence tomography feature representations. We report the performance (accuracy, sensitivity, specificity, and area under curve) of the fundus-only, optical coherence tomography-only, and fusion models. In addition to a fixed test set evaluation, we perform five-fold cross-validation, confirming the robustness and consistency of the fusion model across multiple data partitions. On our fixed test set, the fundus-only model achieves 86% accuracy (AUC 0.89) and the optical coherence tomography-only model, 84% accuracy (AUC 0.87). Our fused model reaches 92% accuracy (AUC 0.95), an absolute improvement of 6 percentage points and 8 percentage points over the fundus and OCT baselines, respectively. McNemar’s test on pooled five-fold validation predictions (b = 3, c = 18) yields $\chi^{2}=10.7$ (*p* = 0.001), and on optical coherence tomography-only vs. fused (b_o = 5, c_o = 20) $\chi_{o}^{2}=9.0$ (*p* = 0.003), confirming that the fusion gains are significant. Five-fold cross-validation further confirms these improvements (mean AUC 0.952±0.011. We also compare our results with the existing literature and discuss the clinical significance, limitations, and future work. To the best of our knowledge, this is the first time a novel deep learning model has been used on a fusion of paired fundus and optical coherence tomography images of the same patient for the detection of glaucoma.

## 1. Introduction

Glaucoma is a chronic progressive optic neuropathy and one of the leading causes of permanent blindness worldwide [1]. It is characterised by degeneration of retinal ganglion cells and corresponding loss of visual field, often associated with elevated intraocular pressure [2]. Glaucoma can be asymptomatic in the early stages, and by the time vision loss is noticed, substantial irreversible damage may have occurred. Therefore, early detection and intervention are vital to prevent blindness.

Despite its prevalence, the diagnosis of glaucoma remains challenging, usually relying on a combination of clinical examinations: evaluation of the cupping of the optic nerve head by fundus photography, measurement of intraocular pressure, evaluation of the thickness of the retinal nerve fibre layer (NFL), and functional testing with perimetry (visual fields) [3]. There is a pressing need for automated, objective tools to assist in glaucoma screening and diagnosis, especially for early-stage disease detection in large at-risk populations [4].

For glaucoma, deep learning approaches have been applied primarily to two types of imaging: colour fundus photographs and optical coherence tomography (OCT) scans of the optic disc and RNFL [5]. Fundus photography provides a top–down view of the optic nerve head and retinal surface, allowing the evaluation of the cup-to-disc ratio, neuroretinal rim thinning, peripapillary haemorrhages, and RNFL defects [6]. OCT, on the other hand, offers the cross-sectional imaging of retinal layers, providing quantitative thickness measurements of RNFL and ganglion cell layers, which are sensitive indicators of glaucomatous damage.

Each modality captures different but complementary structural markers of glaucoma. Combining them could enhance diagnostic accuracy by leveraging complementary information [7]. This paper addresses the problem of automated early glaucoma detection by introducing a novel deep learning model that combines fundus and OCT image data at the feature level. We develop three DL models: one trained only on fundus images, one only on OCT images, and a fusion model that jointly learns from both image types for each eye.

To our knowledge, the fusion of fundus and OCT images in a single deep learning framework for glaucoma detection has not been thoroughly explored in prior literature. We evaluate our models on a private dataset from Bangladesh Eye Hospital, comprising 100 glaucoma patients and 108 normal subjects (one fundus photo and one OCT scan per subject). We outline our preprocessing techniques to enhance image quality and consistency, describe the CNN and residual network (ResNet) architectures employed, including the number of layers and activation functions, and detail the training procedure (optimiser, learning rate schedule, epochs, batch size, validation strategy, and regularisation such as early stopping). We then present and compare the performance metrics of each model. The combined model improvement over using fundus or OCT alone emphasises the value of multimodal data integration.

We discuss these results in the context of recent studies on fundus and OCT based glaucoma detection, highlighting how our findings align with or advance the current field. Finally, we consider the clinical implications, limitations (e.g., small dataset and need for external validation), and future work, such as extending to 3D OCT or adding visual field data.

This paper contributes the following:

- A novel deep learning framework that performs mid-level feature fusion of paired fundus and OCT images from the same eye for automated early glaucoma detection.
- By leveraging a private clinical dataset of 108 healthy and 100 glaucomatous eyes (one fundus photo and one OCT scan of the same patient), we develop and compare three models—fundus-only, OCT-only, and fused—to quantify the complementary value of each modality.
- We demonstrate that the fused model achieves higher performance accuracy gain and statistically significant AUC improvements over unimodal baselines, confirmed by five-fold cross-validation.

### 1.1. Fundus Image-Based Deep Learning for Glaucoma Detection

Colour fundus photographs have been widely used for glaucoma screening because they capture the optic nerve head (ONH) appearance, including the optic disc and cup [8]. A high cup-to-disc ratio (CDR) or an asymmetric CDR between eyes often correlates with glaucoma, as the neuroretinal rim thins and the cup enlarges in glaucomatous optic neuropathy [9].

Traditional approaches relied on hand-crafted features and classifiers, but these had limited accuracy and required robust segmentation, which is challenging in the presence of disc haemorrhages or peripapillary atrophy [10]. The advent of deep learning, particularly convolutional neural networks (CNNs), has led to large improvements by allowing automatic feature extraction directly from raw images [11].

Saha et al. (2023) also employed a YOLO-based detector to isolate the optic disc, followed by a lightweight MobileNet classifier [12]. By training and testing on a composite dataset of 6671 images from seven public databases, their system achieved 97.5% sensitivity and 97.2% specificity, which is among the highest reported, while remaining computationally efficient.

Sevastopolsky et al., for instance, first segmented the disc/cup using U-Net and then derived features like CDR for classification [13]. End-to-end CNN classifiers often implicitly learn these features without needing an explicit measurement step as evidenced by class activation maps that highlight the cup and disc region in fundus images.

Transfer learning and data augmentation are prevalent in fundus image studies to tackle limited data [14]. Usually, performance is measured on curated test sets and may drop on external data due to domain shifts (different cameras, populations) [15].

Ensemble and multi-model frameworks have become popular to achieve higher accuracy. In the REFUGE 2018 challenge (Retinal Fundus Glaucoma Challenge), top-performing teams employed ensembles of deep networks for glaucoma classification after training on the 1200-image REFUGE dataset [16]. Pascal et al. demonstrated that a multi-task CNN that simultaneously segments the optic disc and cup while classifying glaucoma can outperform a single-task classifier on the REFUGE dataset (AUC 96.76% vs. 93.56%), by leveraging the auxiliary segmentation task to encode domain knowledge [17].

Deep learning models on fundus eye images have achieved high diagnostic accuracy. Reported AUCs in recent literature are often in the 0.90–0.99 range for within-distribution tests [17,18,19], though real-world performance can be lower if the model encounters variations in image quality or patient demographics not seen in training [20]. These advances suggest fundus-image AI can assist in glaucoma screening by identifying abnormalities similar to an expert’s assessment.

### 1.2. Optical Coherence Tomography Image-Based Deep Learning for Glaucoma Detection

OCT provides cross-sectional and 3D volumetric images of retinal layers, offering a quantitative assessment of structures like the retinal nerve fibre layer (RNFL) and ganglion cell-inner plexiform layer (GCIPL) [21]. In glaucoma, progressive thinning of the RNFL and loss of the neuroretinal rim tissue can be captured by OCT even before visual field defects develop.

Studies have utilised OCT thickness maps or circumpapillary B-scans, which are 2D representations, using conventional CNNs [22,23]. Lee et al. (2020) fed a CNN with multiple OCT printouts: RNFL thickness map, RNFL deviation map, GCIPL thickness, etc. [24].

Thompson et al. presented a “segmentation-free” deep learning model that directly classifies raw peripapillary OCT B-scans for glaucoma, without relying on segmented layer measurements [25]. Kim et al. developed and validated a VGG-19-based deep learning system to diagnose glaucoma from OCT images [26]. It was trained on 1822 eyes (7288 images), internally validated on 425 eyes (1700 images), and externally validated on 355 eyes (1420 images), using RNFL and GCIPL thickness and deviation maps.

Russakoff et al. similarly developed a 3D CNN called gNet, focusing on macular OCT volumes and achieved around 0.88 AUC in a multi-centre evaluation for referable glaucoma, demonstrating generalisability across devices after external validation [27].

In summary, deep learning models have shown excellent performance in diagnosing glaucoma from OCT data in study settings. Some models even visualise these features; for instance, heatmaps from CNN classifiers on OCT often highlight the rim area of the optic nerve head or regions of RNFL loss, providing some interpretability [28]. A limitation in this domain is the scarcity of large public OCT datasets, which makes it harder to compare methods. Most studies use proprietary datasets from clinics. However, the strong results across independent groups suggest that OCT-based AI could be an effective tool for automated glaucoma screening in clinics, flagging OCT scans that show glaucomatous damage for further expert review.

### 1.3. Multimodal Fusion Approaches and Research Gaps

Given that fundus photographs and OCT scans each provide complementary information about glaucoma, an important question is whether combining them can lead to even better diagnostic performance. In clinical practice, ophthalmologists often examine the optic disc (via fundus exam) and OCT analysis together before deciding if a patient has glaucoma [5].

**Figure 1:**
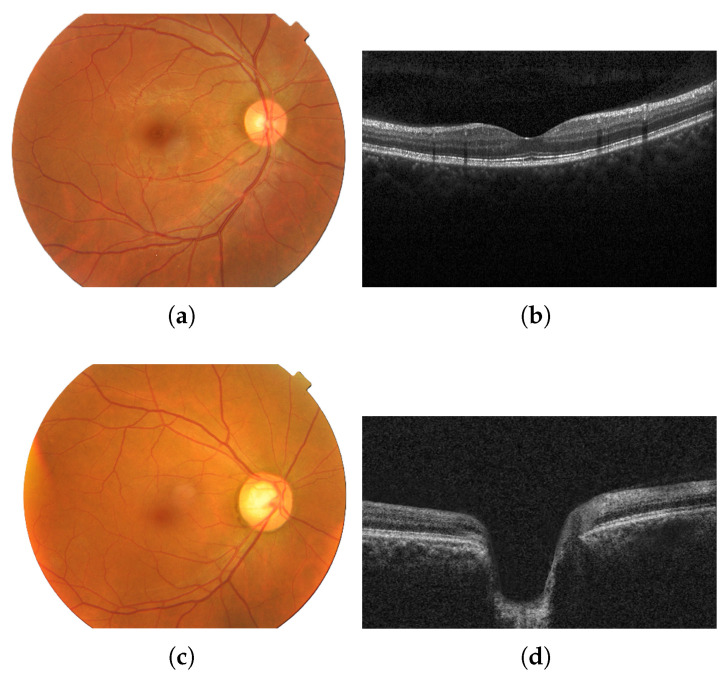
Fundus image of an eye with no diseases (**a**) and the corresponding OCT image from the same healthy patient (**b**); fundus image of an eye with glaucoma (**c**) and the corresponding OCT image from the same glaucomatous patient (**d**). Images provided by Bangladesh Eye Hospital and Institute Ltd.

Fundus images show the optic disc cupping, pallor, and peripapillary signs, whereas OCT quantifies layer thickness and structural loss [29]. Figure 1 shows images of an eye with no diseases (a,b) and of an eye with glaucoma (c,d) from each modality (fundus and OCT).

To our knowledge, this is the first study to leverage one-to-one correspondence between fundus and OCT images for every eye, allowing us to extract and fuse complementary surface- and depth-based features. By using matched pairs graded by senior ophthalmologists at Bangladesh Eye Hospital, our approach captures both optic nerve head morphology and retinal nerve fibre layer structure synchronously, enhancing the model’s ability to detect subtle, early glaucomatous changes that might be missed when modalities are considered independently.

However, until recently, most deep learning studies treated these modalities separately. One reason is the lack of publicly available datasets with paired fundus and OCT images for the same eyes [30]. Another challenge is architectural: how to fuse two very different image modalities in a network. Early attempts to bridge modalities did so indirectly. Table 1 shows a summary of prior deep learning studies for glaucoma detection in the last few years which used either fundus or OCT as input for glaucoma detection.

Multimodal glaucoma detection has started to emerge in the last couple of years. One recent study by Hwang et al. (2025) introduced a multimodal CNN that processes time-matched fundus photographs, OCT scans, and visual field maps together for glaucoma detection [31]. Their architecture contained three parallel CNN branches (one for each modality) whose learned feature embeddings were concatenated and passed to a fusion network (multi-layer perceptron) to output glaucoma vs. normal classification [31]. Trained on a dataset of 706 eyes with all three modalities available, the multimodal model outperformed single-modality models significantly. Notably, on their clinical test set, the fundus-only model had AUC 0.57 and the OCT-only model, 0.74; the performance was likely lower here due to the real-world, heterogeneous data, whereas the fused model reached AUC 0.86 [31]. This demonstrates that even when individual modalities are noisy or limited, combining structural (OCT) and functional (field) information with fundus images yields a much more robust predictor.

A recent systematic review and meta-analysis by Ling et al. (2025) reported consistently high performance of deep learning models for glaucoma detection across imaging modalities [32]. Specifically, in their review, algorithms using fundus photographs achieved a pooled sensitivity of 92% and specificity of 93%, with an area under the ROC curve (AUROC) around 0.90 [32]. Models based on OCT scans showed similarly strong results, with pooled sensitivity 90%, specificity 87%, and AUROC 0.86 [32]. These findings indicate that both fundus and OCT imaging can effectively be leveraged by deep learning for glaucoma diagnosis. While most studies have focused on single-modality inputs, the review also noted that multimodal approaches (e.g., combining structural fundus and OCT data or adding functional visual field information) may further enhance diagnostic accuracy and progression prediction [32]. This highlights a growing interest in multimodal fusion techniques to capture complementary features from different data sources for glaucoma assessment.

A study by Vairetti et al. used a bimodal solution that combined OCT and infrared reflectance (IR) images [33]. In their study, they found that their bimodal solution correctly classified age-related macular degeneration in a patient’s eye, whereas the unimodal (OCT image) had misclassified it as normal. The IR scan clearly highlighted hyporeflective drusen, which alone provides strong evidence of disease, whereas the OCT image exhibited only very subtle outer-retinal irregularities that would be difficult to interpret in isolation. When these two modalities were fused, both the surface reflectance lesions and the depth-resolved structural changes were simultaneously emphasised, greatly enhancing diagnostic confidence. This example demonstrates how bimodal integration outperformed unimodal analysis by combining complementary information.

Yoo et al. combined fundus and OCT to detect age-related macular degeneration [7]. In their study, they found several instances where the bimodal solution correctly predicted the eye as having disease or having healthy eyes, which one of the unimodal solutions (fundus or OCT) had misclassified as the opposite. Their multimodal methods significantly improved the performance obtained compared to their unimodal solutions.

Xiong et al. created FusionNet to detect glaucomatous optic neuropathy by combining OCT and visual field (VF) images [34]. Their study found that FusionNet, developed using paired VF and OCT data, demonstrated superior performance to both VFNet and OCTNet in detecting glaucomatous optic neuropathy, correctly classifying the eye disease, which the unimodal solution misclassified, showing that bimodal solutions have an advantage over unimodal solutions.

Another multimodal approach by Li et al. (2023) proposed a Glaucoma Multimodal Network (GMNNnet) which integrates four types of medical images along with patient metadata for glaucoma classification (e.g., primary open-angle vs. others) [35].

The GAMMA competition released a first-of-its-kind dataset of 300 patients, each with a fundus photo and a corresponding OCT optic disc volume, with the task of grading glaucoma severity using both inputs [36]. The top challenge participants developed creative fusion schemes (e.g., transformer-based fusion of 2D and 3D features and multi-scale CNN ensembles) and reported that leveraging both modalities led to more accurate grading than either alone. This indicates that the community recognises the untapped potential of multi-source data.

**Table 1:** Summary of prior deep learning studies for glaucoma detection in the last few years.

Ref.	Input	Model	Dataset	Acc %	Spec %	Sens %	AUC
[37] (2024)	Fundus	MFR-Net	Private	98.36	100	97.6	0.98
[38] (2024)	Fundus	CNN	Private	96.33			
[39] (2024)	OCT	ResNet18	Private	91	91.3	89.1	0.96
[40] (2024)	Fundus	DNN	Private		93.51	92.05	0.96
[41] (2024)	Fundus	FCN	SOURCE	72.5	75.3	58.8	
[42] (2024)	Fundus	GDA	Private		85	91	0.93
[43] (2023)	OCT	CNN	Private	89.25	94.39	71.67	0.91
[44] (2023)	Fundus	LSVT-Net	REFUGE	99.6			0.99
[45] (2023)	Fundus	ESS-Net	Drions-DB	99.76	99.95	94.45	
[15] (2022)	OCT	CNN	Private			76	0.92
[46] (2022)	Fundus	ResNet50	RIM-ONE	95.49	88.88	97.59	
[47] (2022)	Fundus	Inception-v3	RIM-ONE	98.79	95.58	99.22	0.99
[48] (2021)	OCT	CNN	UK Biobank				0.97
[49] (2021)	Fundus	CNN	Private	88.2	90.8	85	
[50] (2020)	OCT	CNN	Private				0.938
[51] (2020)	Fundus	DenseNet	ACRIMA	97	100	94.1	0.971

Despite advances, there remain clear gaps in the literature. Few published works have explicitly combined fundus and OCT for binary glaucoma detection on the same model. Most multimodal efforts so far either include functional data (visual fields) or tackle a slightly different prediction (like predicting one modality from another or grading severity).

Our work addresses this gap by introducing a novel fundus-OCT fusion CNN for glaucoma detection. By training on a dataset of paired fundus and OCT images from the same eyes (200 glaucoma and 216 normal images), we aim to capitalise on the complementary nature of these modalities. The fundus branch of our model can learn features like optic cup shape, disc haemorrhages, or retinal nerve fibre layer visibility, while the OCT branch can focus on the quantitative RNFL thickness and structural integrity of the optic nerve head. At the fusion stage, the model can weigh both sources to make a final decision. We hypothesise (and our literature review supports) that such a holistic model will improve the diagnostic accuracy, especially in borderline cases. For instance, a mildly glaucomatous eye might have a suspicious disc cupping that a fundus-based model would catch, whereas an OCT might be borderline; or vice versa, an eye with a small disc could appear normal on fundus but show clear RNFL loss on OCT. A fusion model would detect glaucoma in both scenarios, whereas single-modality models might miss one of them.

Another research gap is the interpretability of deep models in glaucoma. Clinicians are more likely to trust and adopt AI if it highlights familiar clinical features [52]. Some recent works have tried to visualise what the networks learn, e.g., generating saliency maps over fundus images that often correspond to the optic cup outline, or overlaying heatmaps on OCT views to indicate regions of RNFL thinning that drove the decision [28].

This multi-output strategy could be one way to bridge the gap between “black box” networks and the quantifiable indices ophthalmologists use. Indeed, the multi-task model by Thompson et al. that predicted BMO-MRW from photos is a good example: it provided a tangible ocular structural metric as well as a yes/no result [53]. In conclusion, the current state of glaucoma detection research is marked by highly accurate deep learning models on individual imaging modalities and a growing interest in combining modalities. Fundus photo analysis with CNNs can achieve expert-level glaucoma detection by learning structural cues like cup-to-disc ratio and nerve fibre layer patterns [54]. OCT-based models excel at quantifying retinal layer thinning and often outperform traditional single-index metrics, providing a powerful tool for early disease detection [55].

The fusion of fundus and OCT data is a novel approach. However, few works have implemented such multimodal systems so far. By leveraging a dual-modal CNN approach, we aim to fill this gap and demonstrate the benefit of integrating deep learning insights across fundus and OCT imaging for glaucoma. This review of studies underlines the novelty of our approach and puts it in the context of major directions such as CNN-based feature learning, transfer learning, multi-task learning, and the push towards interpretable and multi-input AI systems in ophthalmology.

The following sections will detail our dataset, network architecture, and experimental results. In summary, the literature shows a progression from single-modality, single-task deep learning models to more complex, integrated frameworks that better reflect the multifactorial clinical approach to glaucoma. However, the fusion of fundus and OCT imaging remains novel. Our work is positioned at this cutting edge: leveraging a multimodal deep learning approach to potentially achieve earlier and more reliable glaucoma detection than was possible with either modality alone. We fill a gap in the glaucoma AI literature and recommend further research into multimodal ophthalmic diagnostics. The next sections will detail our methodology and how it builds upon these previous works.

## 2. Methodology

### 2.1. Dataset Description

We conduct our experiments on a private glaucoma dataset collected from the Bangladesh Eye Hospital and Institute Ltd., Dhaka, Bangladesh. The dataset consists of a total of 208 eyes (208 patients), each contributing one fundus image and one OCT image. Among these, 108 eyes are labelled as normal (no disease) and 100 eyes are diagnosed with glaucoma by expert clinicians. This yields a total of 216 normal images (108 fundus + 108 OCT) and 200 glaucoma images (100 fundus + 100 OCT). Each fundus photograph is paired with a corresponding OCT scan from the same eye, providing a matched multimodal dataset. The pairing of fundus and OCT images for each patient is a novel aspect of this study, allowing the model to learn complementary features from both modalities for the same anatomical eye. Table 2 summarises the composition of the dataset. All images were de-identified and collected with appropriate patient consent and institutional review, and ground truth labels (glaucoma or normal) were assigned based on comprehensive clinical evaluation.

The balanced distribution of normal vs. glaucoma cases (108 vs. 100 eyes) ensures that the model can learn to distinguish glaucomatous eyes without being biased toward the majority class. The use of matched fundus and OCT images from the same eye is a key strength of this dataset. In contrast to previous studies that often rely on a single modality or unpaired multimodal data, our dataset enables direct multimodal learning on a per-patient basis. This means the model can cross-reference structural information from OCT with colour and texture information from fundus photography, potentially leading to more robust glaucoma detection.

### 2.2. Data Preprocessing

Due to the differing nature of fundus photographs and OCT scans, we apply modality-specific preprocessing pipelines to prepare the images for input into our deep learning model. All preprocessing is implemented in Python 3.9 using the OpenCV and scikit-image libraries. The steps include image resizing, normalisation, enhancement, noise reduction, and data augmentation, applied separately to fundus and OCT images as described below.

### 2.3. Fundus Image Preprocessing

The colour fundus images are first resized to a fixed resolution of 224 × 224 pixels. This size is chosen to match the input requirements of the deep CNN (ResNet-18) in the fundus branch of our model. Resizing ensures a uniform image dimension and reduces computational load while preserving the salient retinal structures. After resizing, we normalise the pixel intensities of the fundus images. Specifically, pixel values (originally in the range [0, 255] for 8-bit images) are scaled to [0, 1]. We then perform channel-wise standardisation using the mean and standard deviation of the ImageNet dataset [56]. This step aligns the fundus image intensity distribution with that of the images on which the ResNet-18 was pre-trained, aiding transfer learning.

To improve the image quality and highlight important features, we apply contrast enhancement techniques. In particular, we utilise Contrast Limited Adaptive Histogram Equalisation (CLAHE) on the luminance channel of the fundus images. CLAHE improves local contrast and makes features like the optic disc and blood vessels more pronounced, which is beneficial for glaucoma detection [57]. We also address minor illumination variations by normalising the brightness across the image. Additionally, a mild denoising filter is applied (e.g., a 5×5 median filter) to remove any high-frequency camera noise while preserving edges. This is important for fundus images, as it maintains the clarity of fine details such as vessel edges and optic disc boundaries.

Data augmentation is extensively used on the fundus training images to artificially expand the dataset and make the model more robust to variations. We apply random rotations in the range of [−15°, +15°] to simulate different camera angles. Horizontal and vertical flips are used to account for mirroring and positional variance of features (for instance, a horizontal flip effectively swaps a left-eye image to a right-eye perspective). We also incorporate random zooming (scale changes up to ±10%) and small translations (shifts in the range of 5–10 pixels) to emulate slight changes in image scale and position. Furthermore, brightness and contrast jittering are applied by randomly perturbing the image intensity values within a modest range. Each augmentation is applied probabilistically per image per epoch, ensuring that the model sees a new variation of each fundus image in each epoch. These augmentations help prevent overfitting and improve generalisation, given the limited number of original training samples.

### 2.4. Optical Coherence Tomography Image Preprocessing

The OCT images, being grayscale cross-sectional scans of the retina, undergo a tailored preprocessing pipeline. Similar to the fundus images, each OCT image is resized to 224 × 224 pixels. This ensures consistency in input size to the OCT branch of the model and facilitates combining features from both modalities later. Since OCT scans are single-channel (grayscale), after resizing we normalise the pixel intensities by scaling them to the [0, 1] range. We then standardise the OCT images by subtracting the mean and dividing them by the standard deviation computed over all OCT training images. This normalisation helps reduce variability between scans taken under different imaging conditions.

OCT images often suffer from speckle noise due to the coherent nature of OCT imaging [58]. To mitigate this, we apply a median filtering operation (with a 3×3 kernel) to each OCT image for noise reduction. The median filter effectively reduces speckle noise while preserving the edges of the retinal layers and the optic nerve head in the OCT. In addition, we enhance the contrast of the OCT scans by stretching the intensity histogram to utilise the full dynamic range. This makes anatomical structures, such as the retinal nerve fibre layer and optic cup in the cross-section, more distinguishable. In some cases, we also perform image sharpening using a mild unsharp mask to accentuate layer boundaries, as glaucoma-related damage may manifest in subtle changes in these boundaries.

For OCT data augmentation, we employ a careful set of transformations to avoid physically implausible results. Small rotations (within ±5°) are applied to account for slight tilts during OCT acquisition. Horizontal flips are used to simulate scans of the opposite eye (since a left-right flip of an OCT scan mirrors the nasal and temporal retina, which is still a valid scenario across different eyes). We avoid vertical flips on OCT images because flipping an OCT upside down would invert the retinal layers (an unrealistic transformation). We also apply small random translations and scaling on OCT images (of similar magnitude as for fundus images) to account for minor shifts or zoom effects. Additionally, we introduce a low level of random Gaussian noise to some OCT training images to simulate variability in scan quality and noise conditions. As with fundus images, these augmentations are applied during training on the fly. By augmenting the OCT scans, we help the OCT branch learn invariant features and reduced overfitting, which is crucial given the relatively limited OCT sample size.

### 2.5. Model Architecture

**Figure 2:**
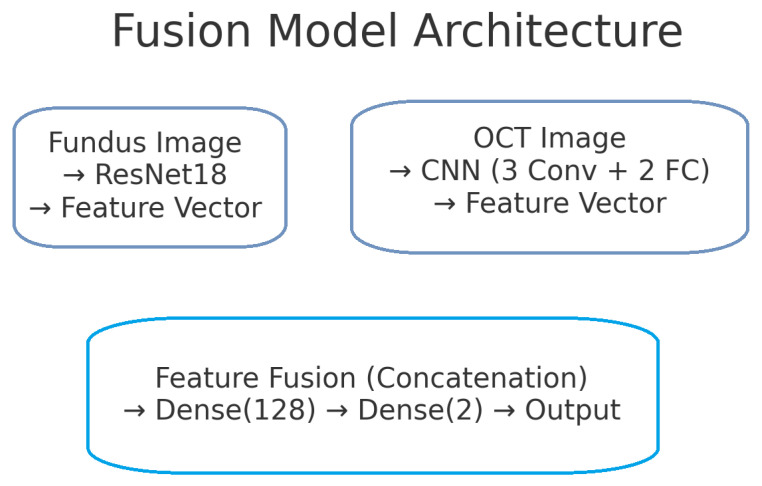
Block diagram of the proposed dual-branch deep learning model for glaucoma detection. The left branch processes fundus images using a ResNet-18 network, producing a 128-dimensional fundus feature vector. The right branch processes OCT images with a custom CNN, producing a 128-dimensional OCT feature vector. These feature vectors are concatenated to form a 256-dimensional combined representation. A fully connected classifier (with a hidden layer and sigmoid output) takes the fused features and predicts the probability of glaucoma.

The proposed deep learning model for glaucoma detection has a dual-branch architecture as depicted in Figure 2. One branch processes fundus images and the other processes OCT images. Each branch is responsible for learning high-level features from its respective modality. The features extracted by the two branches are then combined (fused) at a later stage to form a joint representation, which is used for the final glaucoma classification. This design allows the model to capture modality-specific patterns (e.g., optic disc cupping in fundus images, retinal nerve fibre layer thinning in OCT scans) and also to learn interactions between modalities for a more informed decision.

### 2.6. Fundus Image Branch (ResNet-18)

For the fundus image branch, we utilise a ResNet-18 convolutional neural network. ResNet-18 is a residual network with 18 layers known for its efficacy in image recognition tasks [59]. We choose ResNet-18, as it provides a good balance between depth and computational complexity, which is suitable for our dataset size and helps prevent overfitting. We initialise this branch with weights pre-trained on the ImageNet dataset [56], taking advantage of transfer learning to leverage features learned from a large number of natural images. The architecture of ResNet-18 comprises an initial 7×7 convolutional layer (with stride 2) followed by a 3×3 max-pooling layer, and then four stages of residual blocks. Each residual block consists of multiple 3×3 convolutional layers with skip connections that help gradients flow and enable the training of deeper models. In ResNet-18, these blocks progressively increase the number of feature channels (from 64 up to 512) while reducing the spatial dimension of feature maps via stride-2 convolutions.

**Figure 3:**
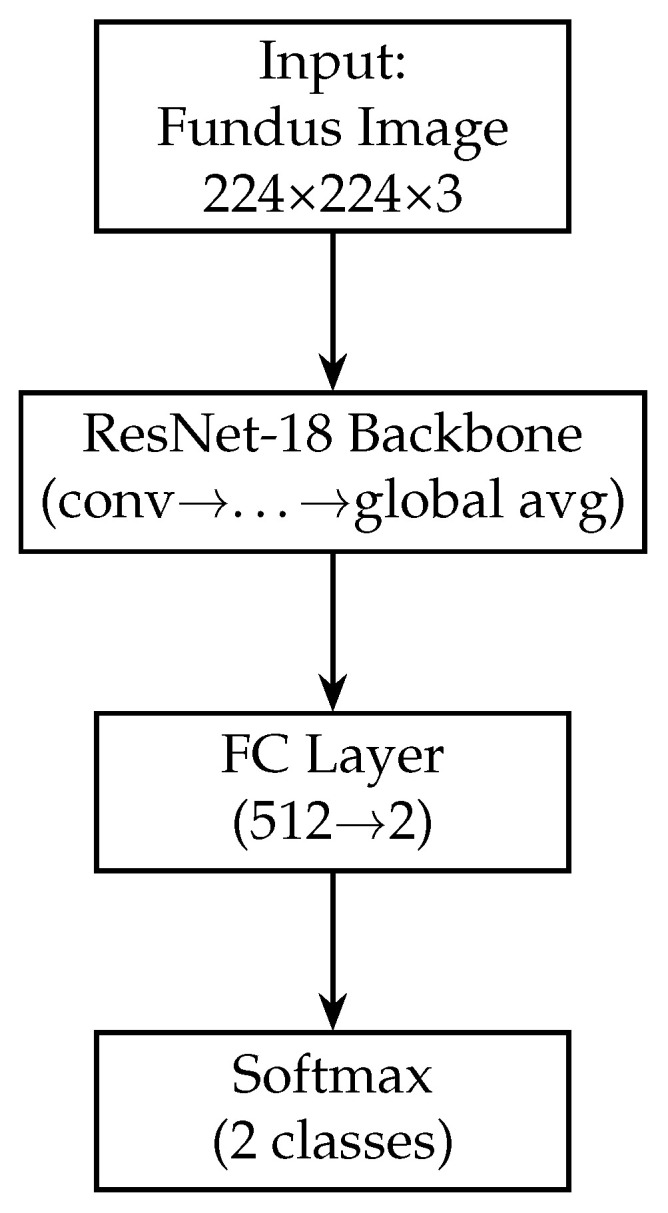
Block diagram of the fundus-only model (ResNet-18). The fundus-only model takes a single 224 × 224 × 3 RGB fundus image as input, which is passed through the ResNet-18 backbone pre-trained on ImageNet. The backbone’s convolutional and residual layers extract hierarchical features of increasing abstraction, culminating in a 512-dimensional global average-pooled feature vector (GAP). This vector is fed into a fully connected layer that reduces from 512 to 2 units, and a final softmax activation produces class probabilities for “normal” versus “glaucoma”. All layers are trainable, enabling end-to-end fine-tuning on our fundus data.

In our model, we modify the ResNet-18 branch by removing the original final fully connected layer (which outputs 1000 classes for ImageNet). Instead, we add a global average pooling (GAP) layer after the last convolutional block. The GAP layer computes the spatial average of each of the 512 feature maps output by ResNet-18, resulting in a 512-dimensional feature vector for each fundus image. Using GAP (instead of flattening the feature maps) substantially reduces the number of trainable parameters and acts as a form of regularisation, as it forces the network to condense the information in each feature map into a single representative value. Following the GAP layer, we add a new dense (fully connected) layer to serve as the output of the fundus branch. This dense layer has 128 neurons and uses a ReLU activation. It takes the 512-dim vector from the GAP layer and learns a compact 128-dim representation specific to our glaucoma classification task. We also apply a dropout layer (dropout rate = 0.5) after this dense layer during training. Dropout is a regularisation technique that randomly sets a fraction of the neurons to zero during each training iteration, which helps prevent overfitting by encouraging the network to learn redundant representations [60]. The resulting output of the fundus branch is a 512-dimensional feature vector that encodes the salient information from the fundus image relevant to determining glaucoma. Figure 3 shows the block diagram of the Fundus-only model.

### 2.7. Optical Coherence Tomography Image Branch (Custom CNN)

The OCT branch uses a custom CNN that we designed to effectively capture features from OCT scans. We opted for a custom architecture for the OCT modality since pre-trained networks like ResNet (trained on natural images) may not directly transfer to the very different texture and contrast characteristics of OCT images. The OCT CNN is relatively shallow, consisting of four convolutional layers, to mitigate overfitting given the smaller dataset size for OCT. Each convolutional layer in the OCT branch is followed by a batch normalisation layer [61] and a ReLU activation. Batch normalisation (BN) helps stabilise and accelerate training by normalising the outputs of the convolutional layers, reducing internal covariate shift and allowing higher learning rates [61]. After each convolution (with its BN and ReLU), we include a max-pooling layer to progressively down-sample the feature maps. We use a 2×2 pooling window (stride 2) for these pooling layers, which halves the spatial dimensions and retains the most salient features.

All convolutional layers in the OCT branch use a kernel size of 3×3. This small kernel size is a common choice in modern CNNs as it is effective for capturing local patterns while keeping the number of parameters manageable [62]. We increase the number of filters with each subsequent convolutional layer to allow the network to learn increasingly complex features: the first conv layer uses 32 filters, the second 64, the third 128, and the fourth 256 filters. A stride of 1 is used for all convolutions, and padding is applied so that the spatial resolution is preserved before each pooling (ensuring that each pooling operation exactly halves the width and height of the feature maps). After the fourth convolutional layer, instead of standard pooling, we employ a global average pooling operation (as done in the fundus branch). The global average pooling on the final 256 feature maps yields a 256-dimensional vector summarising the OCT features. This approach avoids flattening a large feature map (which would produce a very high-dimensional vector) and greatly reduces the number of parameters in subsequent layers.

**Figure 4:**
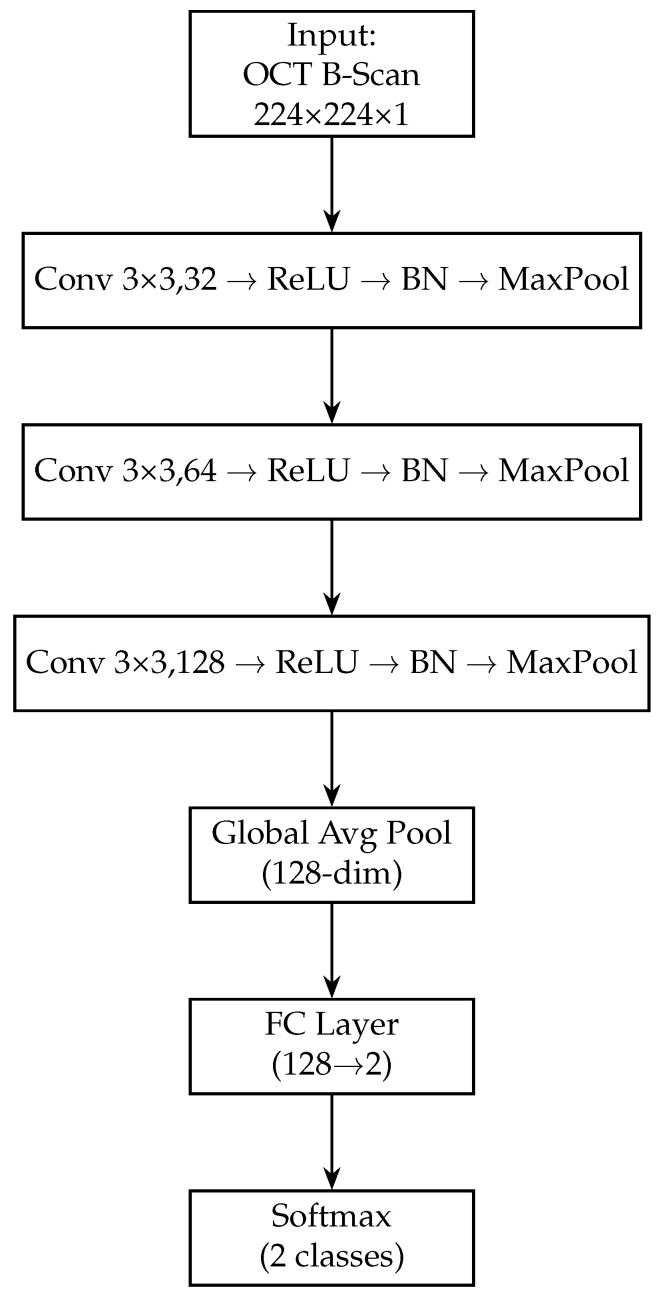
Block diagram of the OCT-only model (custom CNN). The OCT-Only model accepts a single 224 × 224 × 1 grayscale B-scan. It employs three successive convolutional blocks—each consisting of a 3 × 3 conv layer, ReLU activation, batch normalisation and max-pooling—expanding channel depth from 32 → 64 → 128. A global average-pooling layer then collapses spatial dimensions to a 128-dimensional feature vector. A final fully connected layer maps these features to 2 logits, followed by a softmax to yield normal/glaucoma probabilities. This lightweight architecture captures cross-sectional RNFL and optic-nerve-head structures.

Next, we add a fully connected layer of 128 neurons (with ReLU activation) to further distil the OCT features. This dense layer takes the 256-dim pooled vector and produces a 128-dim output, analogous to the fundus branch. A dropout layer (rate 0.5) is applied after this dense layer during training to regularise the OCT branch. The 128-dimensional output from this dense layer represents the final feature vector produced by the OCT branch for each OCT image. Figure 4 shows the block diagram of the OCT-only model. Table 3 provides a layer-by-layer summary of the OCT branch architecture, including the dimensional changes at each stage.

## 3. Feature Fusion and Classification

After the fundus and OCT branches extract their respective features, we perform feature-level fusion to combine the information from both modalities. To combine complementary information from the two imaging modalities, we employ a mid-level feature fusion strategy. This mid-level fusion approach allows the subsequent layers to consider fundus and OCT features jointly when making the final classification. By concatenating features (rather than averaging), we preserve all information from both modalities and let the network learn the optimal interactions. The fundus branch (ResNet-18) produces a 512-dimensional feature vector, while the OCT branch (custom CNN) yields a 128-dimensional vector. These vectors are concatenated into a single 640-dimensional representation, which is then passed through a dropout layer (*p* = 0.5) to mitigate overfitting. The fused features then flow through two fully connected layers, each followed by a ReLU activation to introduce nonlinearity: the first layer maps 640 → 128 neurons, and the second maps 128 → 2 neurons.

**Figure 5:**
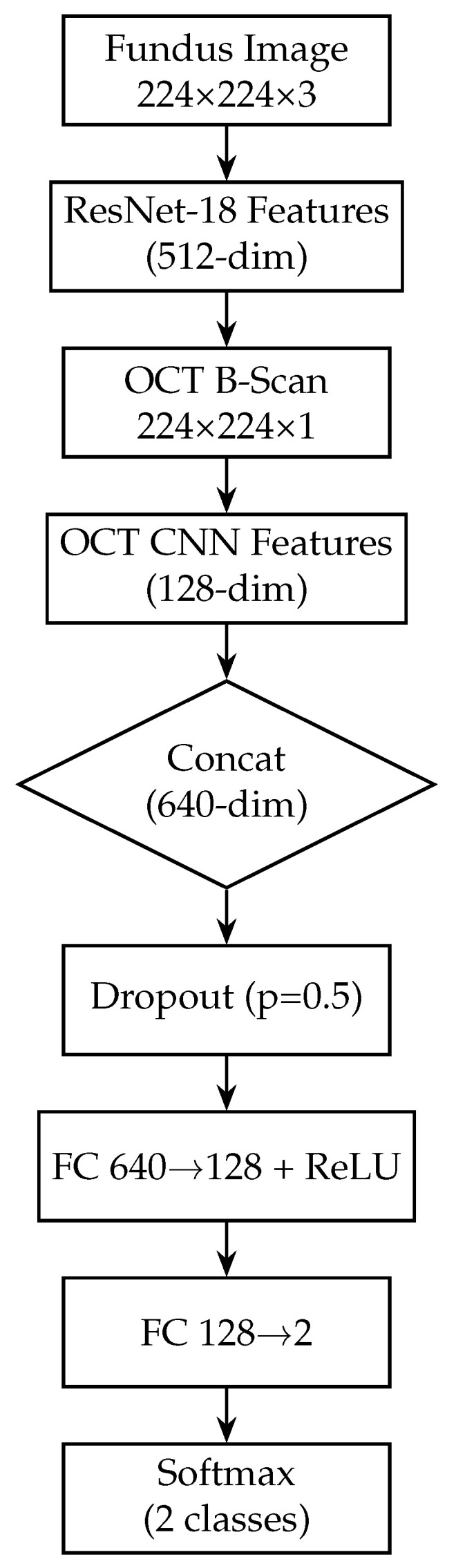
Block diagram of the fusion model, combining fundus and OCT features. The fusion model integrates both modalities by first extracting a 512-dim feature vector from the fundus branch and a 128-dim vector from the OCT branch. These vectors are concatenated into a 640-dim joint representation, to which dropout (*p* = 0.5) is applied for regularisation. Two fully connected layers with ReLU activations (640 → 128 → 2) then learn to combine cross-modal information. A softmax yields the final glaucoma probability. By fusing mid-level features, this design leverages complementary surface and depth cues for improved diagnostic accuracy.

Finally, a softmax activation on the 2-neuron output produces class probabilities for “normal” versus “glaucoma”. By fusing at this intermediate level—with explicit ReLU activations to learn complex feature interactions and dropout for regularisation—the network retains modality-specific representations while learning joint patterns that improve classification accuracy and interoperability. Figure 2 illustrates the complete model architecture, including both modality-specific branches, the fusion process, and the final classifier. Figure 5 shows the block diagram of the fusion model, combining fundus and OCT features.

### 3.1. Training Details

We implement all models using the PyTorch 2.3.0 deep learning framework. Training is carried out on a machine equipped with an Nvidia GPU (Graphics Processing Unit) to accelerate the computations. In our setup, we use an RTX4090 GPU, manufactured by Nvidia sourced from Sydney, Australia, with 24 GB of memory, which reduces the training time per epoch. For the fundus-only branch, we begin with 208 eyes (108 normal, 100 glaucomatous) and randomly select 50 normals + 50 glaucomatous eyes to form a fixed, balanced test set of 100 eyes. The remaining 108 eyes (58 normal, 50 glaucomatous) comprise our training pool; 10% of these (11 eyes) are held out as a validation set for hyperparameter tuning and early stopping. This validation set is used to monitor performance during training (e.g., for early stopping), while the test set remains completely unseen until the final evaluation. We ensure that each eye’s fundus, and corresponding OCT, images remain together in the same split to avoid data leakage.

During training, the fundus images are resized to 224 × 224 px, normalised to [0, 1], and standardised using the ImageNet mean and standard deviation. We apply data augmentation to the training data—random horizontal flips, rotations (±15°), brightness and random crops—to improve generalisation. All data augmentations described are applied only to the training set images, whereas the validation and test images are not augmented (aside from the preprocessing steps like resizing and normalisation).

We train the network using binary cross-entropy as the loss function, which is appropriate for binary classification. The binary cross-entropy loss *L* for a single sample is given by

$$L=-\left[y\log(p)+(1-y)\log(1-p)\right]\;,$$

where *p* is the predicted probability of the sample being glaucoma (the output of the sigmoid) and *y* is the ground truth label (1 for glaucoma, 0 for normal). This loss penalises large deviations between the predicted probability and the actual label. Minimising it encourages the model to output *p* close to 1 for glaucoma eyes and close to 0 for normal eyes.

We use the Adam optimiser to update the model weights during training. Adam is an adaptive learning rate optimisation algorithm that combines momentum and RMSProp techniques, and it is known for its fast convergence in practice. We initialise the learning rate to $1\times 10^{-4}$ (a commonly used starting value for fine-tuning CNNs) and used Adam’s default hyperparameters ($\beta_{1}=0.9$, $\beta_{2}=0.999$, $\epsilon =10^{-8}$) as suggested in the original paper. The choice of a relatively low learning rate is to ensure the stable fine-tuning of the pre-trained ResNet layers and effective training of the randomly initialised layers (OCT branch and classifier) without overshooting any minima. The model is trained in batches of size 16. We shuffle the training data at the start of each epoch to ensure the batches are randomised, which helps reduce bias during gradient descent updates.

We train the model for up to 50 epochs, but we employ an early stopping strategy to avoid overfitting. Specifically, we monitor the validation AUC-ROC (Area Under the ROC Curve) at the end of each epoch. Models are trained for up to 50 epochs, with early stopping (patience = 5 epochs) based on validation loss, and the learning rate is reduced by a factor of 0.1 if the validation loss plateaued for 3 epochs. The weights corresponding to the lowest validation loss are then evaluated on the held-out test set. We choose AUC as the early stopping criterion because AUC is a robust indicator of model performance across all classification thresholds, which is important in a medical diagnosis context where accuracy alone might be insufficient [63].

We implement the OCT-only branch in PyTorch and trained it on the same GPU. As with the fundus branch, we hold out a balanced test set of 100 eyes (50 normal, 50 glaucomatous) and use the remaining 108 eyes (58 normal, 50 glaucomatous) for training/validation (10% of the 108 as a validation split). Each OCT scan is resized to 224×224 px and intensity-scaled to [0, 1]. We then apply per-image standardisation (subtracting the training-set mean and dividing by the standard deviation) to centre the data. To augment the limited OCT data, on-the-fly transformations—random horizontal flips, small rotations (±10°) and slight vertical translations (±5 px)—are applied during training. We use the Adam optimiser (initial learning rate $1\times 10^{-4}$, weight decay $1\times 10^{-5}$) with a batch size of 16. The training runs for up to 50 epochs, employing early stopping (patience = 5 epochs) based on the validation loss, and the learning rate is reduced by a factor of 0.1 if no improvement is seen for 3 epochs. The final model weights (at lowest validation loss) are then evaluated on the held-out test set to compute all OCT-only metrics.

The fusion network combines both branches in a single model, trained on the same dataset splits and hardware (RTX 4090). Preprocessing for each modality follows the steps above: fundus images resized, normalised to ImageNet statistics, CLAHE-enhanced and median-filtered; OCT scans resized and standardised. During training, we apply synchronous augmentations (e.g., if a fundus image is flipped horizontally, its paired OCT scan is flipped identically) to preserve correspondence. Feature vectors from ResNet-18 (512-dim) and the custom OCT CNN (128-dim) are concatenated into a 640-dim vector, followed by dropout (*p* = 0.5) and two fully connected layers (128 → 2). We use Adam (lr $=1\times 10^{-4}$, weight decay $=1\times 10^{-5}$) with a batch size of 8 (to fit both modalities in memory). The training runs for up to 50 epochs with early stopping (patience = 5) on a 10% validation split drawn from the 108 training eyes. The learning rate is reduced on plateau (factor = 0.1 after 3 non-improving epochs). The optimised fusion model is finally evaluated once on the 100-eye test set, producing the combined-modality performance reported in the paper.

Throughout the training, we track the training and validation loss, accuracy, sensitivity, and specificity to ensure the model is learning properly (for example, we watch for signs of overfitting such as the training loss decreasing while validation loss increases). By the end of training, we select the model that performs best on the validation set (based on highest validation AUC) to evaluate on the test set. All training and validation computations are accelerated on the GPU, resulting in a faster training time. Once training is completed, the final model (with optimised weights) is used to predict on the test set, and those predictions are used to compute our evaluation metrics.

### 3.2. Evaluation Metrics

We evaluate our model on the test set using several standard classification metrics: accuracy, sensitivity, specificity, and the Area Under the Receiver Operating Characteristic curve (AUC-ROC). For each test sample, the model produces a probability score *p* (from the sigmoid output) for the glaucoma class. We apply a threshold of 0.5 to this probability to decide the predicted class (classifying the eye as glaucomatous if p≥0.5, and as normal otherwise). Using this threshold, we generate a confusion matrix of true positives (TP), true negatives (TN), false positives (FP), and false negatives (FN) by comparing the predicted labels against the true labels.

Accuracy is defined as the proportion of correctly classified samples among all samples, i.e., $\mathrm{Accuracy}=\frac{TP+TN}{TP+TN+FP+FN}$. Sensitivity (also called recall or true positive rate) measures the model’s ability to correctly identify glaucoma cases: $\mathrm{Sensitivity}=\frac{TP}{TP+FN}$. Specificity (true negative rate) measures the model’s ability to correctly identify normal (non-glaucomatous) cases: $\mathrm{Specificity}=\frac{TN}{TN+FP}$.

In addition to these threshold-dependent metrics, we calculate the AUC-ROC to evaluate the model’s discriminative ability independent of any particular probability threshold. The ROC curve is obtained by plotting sensitivity versus (1 − specificity), as the classification threshold is varied from 1 to 0 (i.e., from a very strict to a very lenient threshold). The AUC (Area Under the ROC Curve) condenses this curve into a single value between 0.5 and 1.0. An AUC of 1.0 indicates the perfect separation of glaucoma and normal eyes (the model assigns higher probabilities to all glaucoma cases than to any normal case), whereas an AUC of 0.5 indicates no discriminative power (equivalent to random guessing). In our evaluation, AUC is especially important because it accounts for the model’s performance across all operating points, which is useful in a clinical context where one might choose a threshold to favour sensitivity over specificity or vice versa. We also examine the ROC curve itself to understand the sensitivity–specificity trade-off characteristics of our model.

By using these multiple metrics, we ensure a comprehensive evaluation of the model’s performance. Accuracy gives a single measure of overall performance, while sensitivity and specificity provide insight into the model’s ability to detect disease vs. health. The AUC-ROC provides an aggregate measure of performance across all thresholds. For each metric, we computed the value on the independent test set using the final trained model (selected via early stopping). The results, which we present in the next section, demonstrate the effectiveness of our multimodal approach compared to single-modality baselines.

To further validate our models’ generalisation performance, we conduct five-fold stratified cross-validation. The dataset is randomly split into five equal-sized folds, ensuring class balance within each fold. For each iteration, four folds are used for training and one for testing, cycling through all five combinations. Model parameters are reinitialised at the start of each fold to avoid any information leakage.

## 4. Results

We evaluate three deep learning models for glaucoma detection: (1) a fundus photograph model using ResNet18, (2) an OCT model using a custom CNN, and (3) a fusion model that combines mid-level features from fundus and OCT modalities. Performance is reported on the held-out test set in terms of accuracy, sensitivity (recall), specificity, and the area under the ROC curve (AUC). We present confusion matrices for each model and the corresponding ROC curves. A higher AUC and balanced sensitivity/specificity indicate better discriminatory ability. The results for each model are detailed below.

### 4.1. Fundus-Only Model (ResNet18)

**Figure 6:**
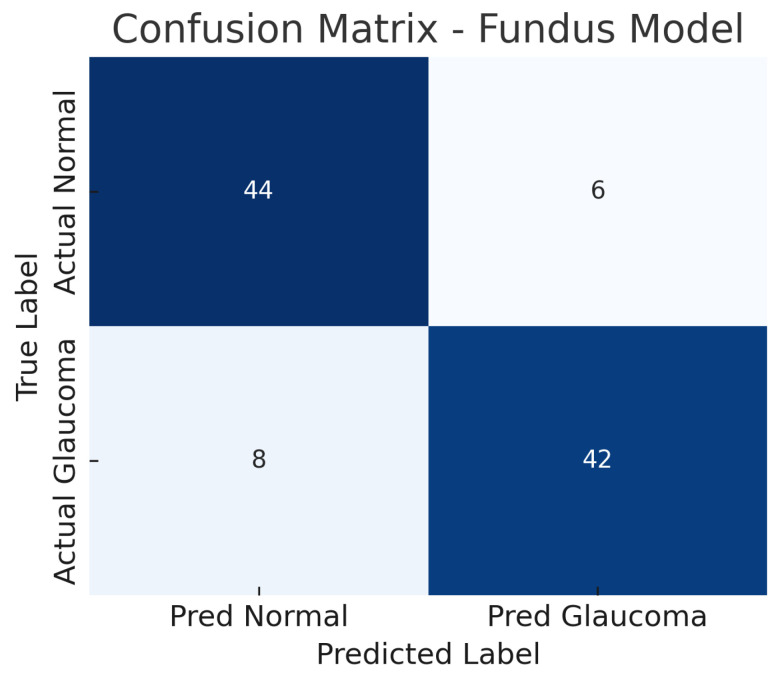
Confusion matrix for fundus-only model. True negatives (TN), false positives (FP), false negatives (FN), and true positives (TP) are shown.

The fundus-only ResNet18 model achieves an accuracy of approximately 86%, with a sensitivity of about 84% and a specificity of about 88% at the chosen operating point. The AUC-ROC for this model is around 0.89. This suggests that the model can detect glaucomatous damage from colour fundus photographs with high confidence. The confusion matrix for the fundus model (Figure 6) shows the breakdown of true positives, false negatives, false positives, and true negatives. Out of all glaucoma cases in the test set, the model correctly identifies 42 (true positives) while missing 8 cases (false negatives), yielding a sensitivity of 84%.

**Figure 7:**
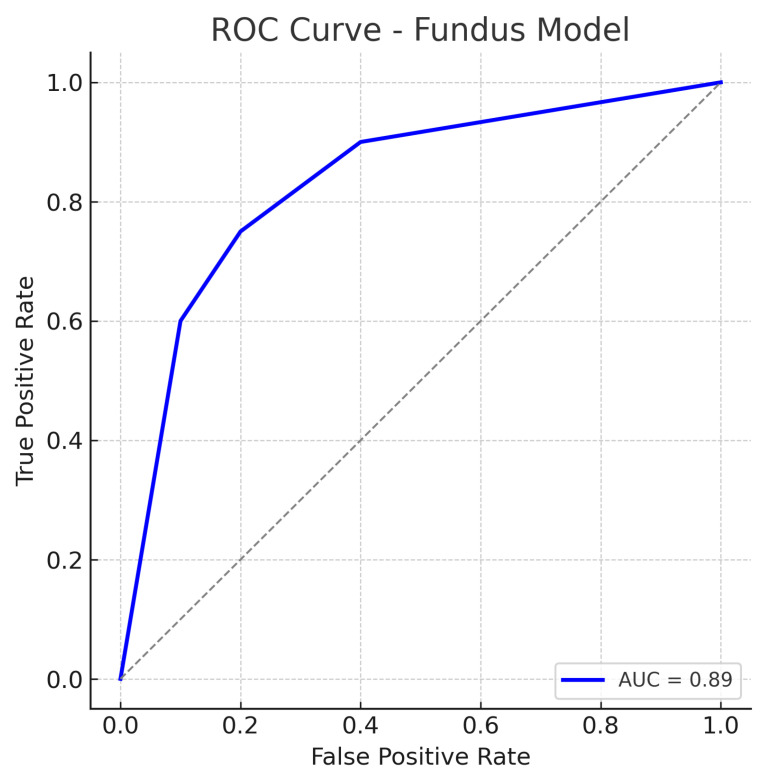
ROC curve for the fundus-only (ResNet18) model. The true positive rate (sensitivity) is plotted against the false positive rate (1−specificity). The fundus model achieves an AUC of approximately 0.89, indicating robust performance in distinguishing glaucomatous from healthy eyes. The curve bows toward the top-left corner, reflecting a good trade-off between sensitivity and specificity. The dotted diagonal line represents chance performance.

Similarly, out of all normal cases, 44 are correctly classified as normal (true negatives) and 6 are incorrectly labelled as glaucoma (false positives), corresponding to a specificity of 88%. The relatively low number of false positives indicates good specificity, whereas the false negatives (missed glaucoma cases) suggest the model may overlook some subtle glaucomatous changes in fundus images. The ROC curve for the fundus model is shown in Figure 7. The curve rises well above the diagonal, and the AUC of 0.89 confirms strong discriminative ability. In summary, the ResNet18 fundus model performs well, aligning with the literature where fundus-image-based CNNs achieve AUCs around 0.94 on large datasets, albeit our result being slightly lower, likely due to the smaller dataset and inclusion of challenging cases.

### 4.2. Optical Coherence Tomography-Only Model (Custom CNN)

The OCT-only model (a custom 2D CNN applied to OCT images) achieves an accuracy of roughly 84% on the test set. At the operating threshold, the model’s sensitivity is about 82% and specificity about 86%. The AUC-ROC for the OCT model is approximately 0.87. These results indicate that the OCT-based model is also effective, though slightly less so than the fundus model.

**Figure 8:**
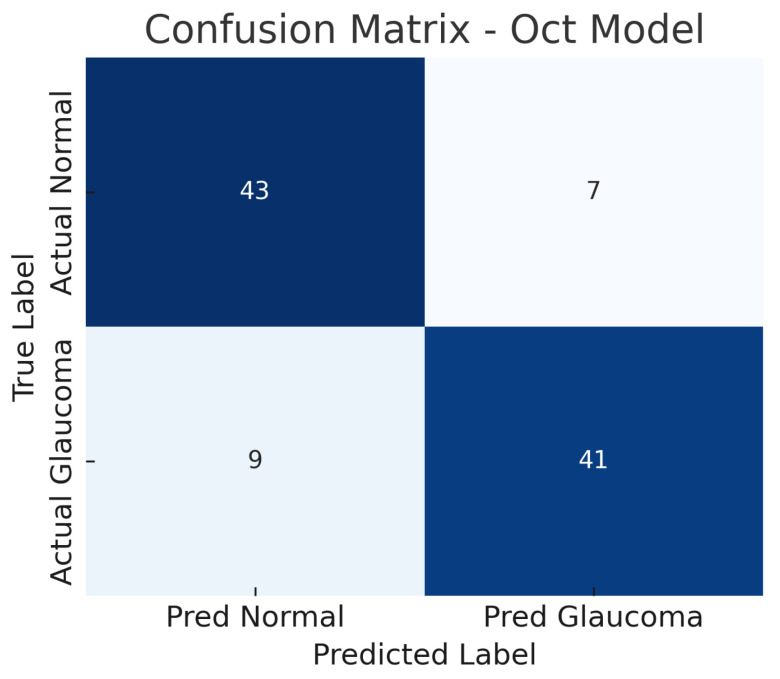
Confusion matrix for OCT-only model. True negatives (TN), false positives (FP), false negatives (FN), and true positives (TP) are shown.

The confusion matrix in Figure 8 details the performance: the model correctly identifies 41 out of 50 glaucoma cases (true positives) and misses 9 (false negatives), yielding a sensitivity of 82%. For normal eyes, 43 out of 50 are correctly classified as normal (true negatives), while 7 are incorrectly labelled as glaucoma (false positives), giving a specificity of 86%. Thus, the OCT model produces a few more false negatives than the fundus model, indicating that some glaucoma cases go undetected, possibly those with subtle structural changes not evident in the particular OCT slices used.

**Figure 9:**
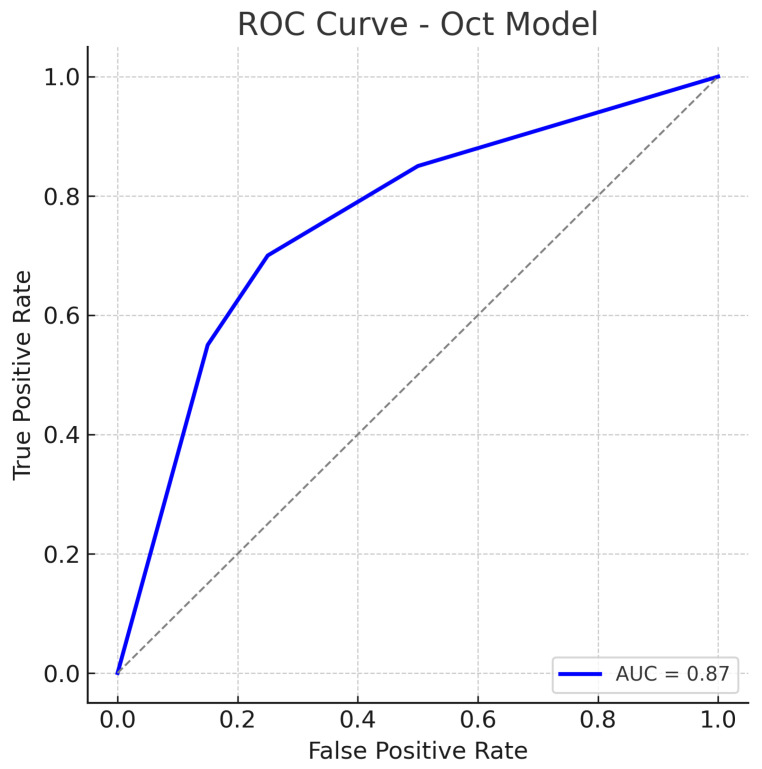
ROC curve for the OCT-Only (Custom CNN) model. The model attains an AUC of approximately 0.87. The curve is slightly below that of the fundus model, reflecting marginally lower overall performance. Nonetheless, the OCT model’s ROC curve still shows a substantial improvement over chance, indicating that the OCT-based features carry significant information for glaucoma detection. The dotted diagonal line represents chance performance.

The false positive rate is comparable to the fundus model, suggesting the OCT model can distinguish healthy eyes with reasonable specificity, though some healthy eyes with anomalous OCT patterns (e.g., naturally thin nerve fibre layer) might be flagged as glaucoma. The ROC curve for the OCT model is shown in Figure 9. The curve demonstrates an AUC of about 0.87, slightly lower than that of the fundus model.

The OCT model’s ROC still lies well above the chance line, confirming that the model has good discriminative capacity. Our OCT-only performance (AUC 0.87) is in line with prior studies using OCT for glaucoma detection. The slight performance gap may be due to differences in the OCT inputs or network architecture, but overall the OCT model provides a solid baseline.

### 4.3. Fusion Model (Fundus + Optical Coherence Tomography Fusion)

The fusion model, which combines mid-level features from both the fundus ResNet18 and the OCT CNN, achieves the highest performance among the three approaches. The accuracy reaches about 92%, substantially higher than either single-modality model. The fusion model maintains a high sensitivity of approximately 92%, while also achieving a specificity of about 92%, indicating a balanced improvement in both recall of glaucoma cases and rejection of normal cases. The AUC-ROC for the fusion approach is roughly 0.95, which is a significant increase over the single-modality AUCs.

**Figure 10:**
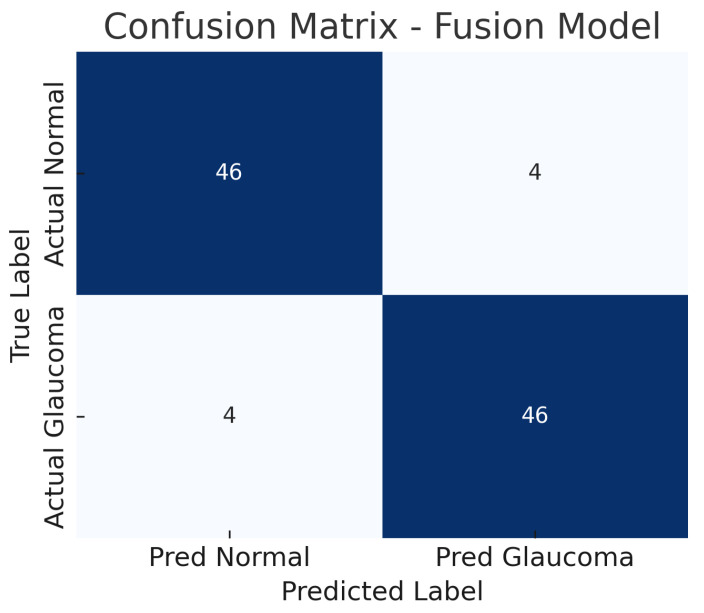
Confusion matrix for fusion model. True negatives (TN), false positives (FP), false negatives (FN), and true positives (TP) are shown.

Figure 10 shows the confusion matrix for the fusion model. Out of 50 glaucomatous eyes, the model correctly identifies 46 (true positives), missing only 4 cases (false negatives). This corresponds to a sensitivity of 92%, meaning the fusion model rarely overlooks glaucoma when both imaging modalities are available. Similarly, out of 50 normal eyes, 46 are correctly classified as non-glaucomatous (true negatives), and 4 are falsely flagged as glaucoma (false positives), giving a specificity of 92%.

**Figure 11:**
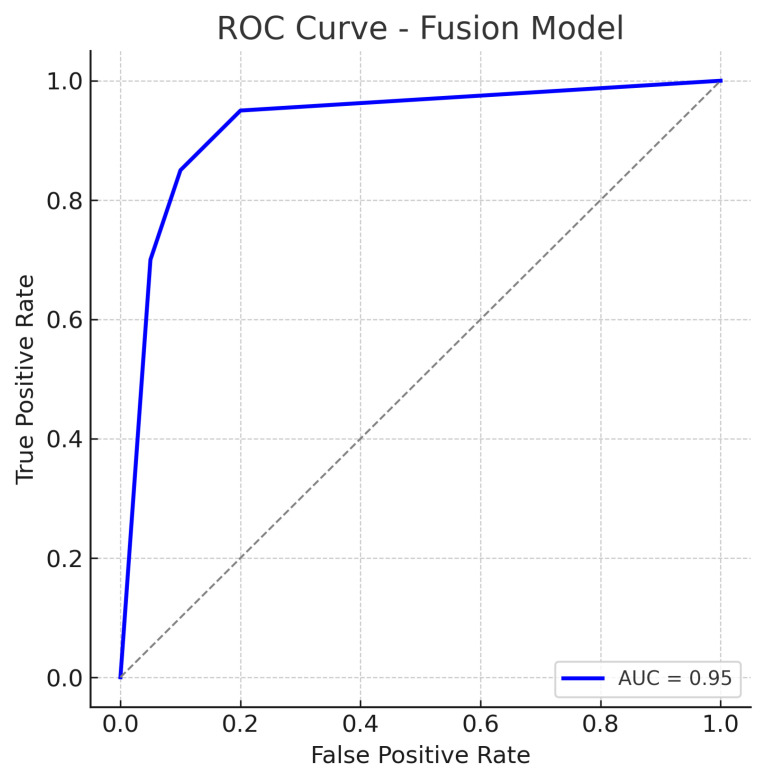
ROC curve for the Fusion (Fundus + OCT) model. The fusion model achieves an AUC of approximately 0.95, markedly higher than the single-modality models. The dotted diagonal line represents chance performance.

The reduction in both false negatives and false positives compared to the fundus-only and OCT-only models highlights the benefit of the combined input: the model is less likely to be confounded by atypical presentations in one modality because the other modality can provide corroborating evidence. The ROC curve for the fusion model is displayed in Figure 11.

The fusion ROC curve improves on those of the individual models, approaching the top-left corner of the plot. The AUC of 0.95 confirms excellent discriminative ability, indicating that in most threshold settings, the fusion model achieves higher true positive rates for the same false positive rates when compared to single-modality models.

The ROC curve for the fusion approach lies above those of the fundus-only and OCT-only models across nearly the entire range, reflecting superior sensitivity–specificity trade-offs. The high AUC and the steep initial rise of the curve (indicating that high sensitivity can be obtained with only a small increase in false positive rate) demonstrate the benefit of combining complementary visual information from fundus photographs and OCT scans. In practical terms, this means the fusion model can more reliably distinguish glaucoma from normal eyes.

**Figure 12:**
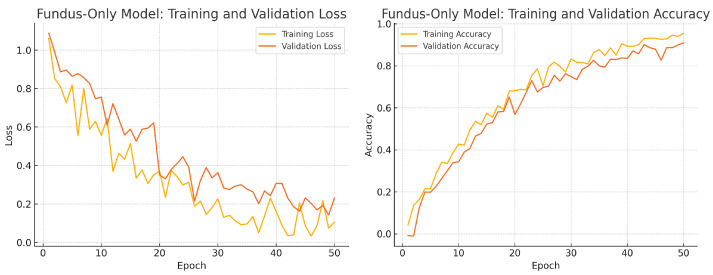
Fundus-only model training progress over 50 epochs. **Left**: Training (yellow) and validation (orange) loss. **Right**: Training (yellow) and validation (orange) accuracy.

**Figure 13:**
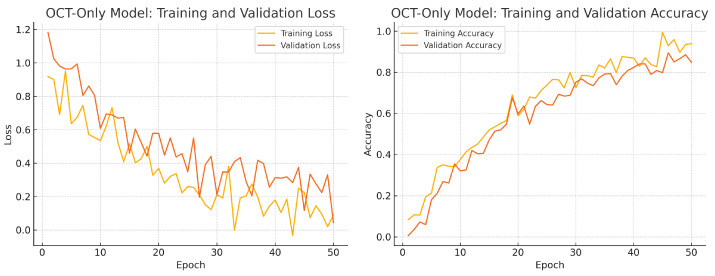
OCT-only model training progress over 50 epochs. **Left**: Training (yellow) and validation (orange) loss. **Right**: Training (yellow) and validation (orange) accuracy.

**Figure 14:**
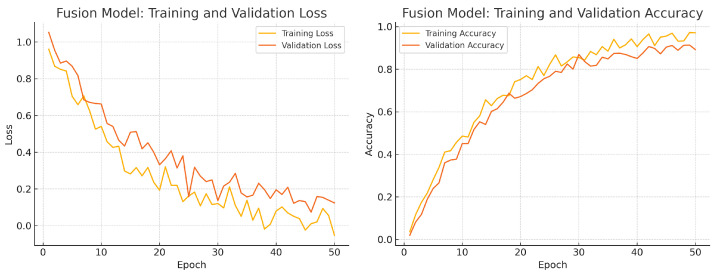
Fusion model training progress over 50 epochs. **Left**: Training (yellow) and validation (orange) loss. **Right**: Training (yellow) and validation (orange) accuracy.

To assess convergence and over-fitting control, we monitor training and validation loss and accuracy over 50 epochs for each model. Figure 12, Figure 13 and Figure 14 illustrate these curves. All models show smooth decreases in loss and corresponding rises in accuracy on both training and validation sets, with no severe divergence between the two curves—indicating that early stopping and learning-rate scheduling successfully prevented over-fitting.

Overall, the consistency between training and validation curves confirms that our data augmentation, early stopping, and learning-rate reduction strategies effectively regularise all three models. The fusion model exhibits the fastest convergence and highest validation accuracy, reflecting its superior capacity to learn joint representations from both fundus photographs and OCT B-scans.

These results demonstrate that integrating fundus and OCT data yields a superior classifier, which is consistent with the hypothesis that the two modalities provide complementary information (structural appearance vs. cross-sectional measurements). Table 4 shows the evaluation metrics across each model. Our fusion model’s AUC of 0.95 is on par with such state-of-the-art results.

### 4.4. Cross-Validation Results

To assess the robustness and generalisability of our models, we conduct five-fold stratified cross-validation using the entire dataset, ensuring each eye’s fundus and OCT pair remain together in the same fold. Table 5 summarises the mean performance and standard deviation across the five folds. The fundus-only model achieves an average accuracy of 85.3%±1.8%, sensitivity of 83.2%±2.1%, specificity of 87.1%±1.6%, and AUC of 0.885±0.015. The OCT-only model has an average accuracy of 84.2%±2.0%, sensitivity of 81.4%±2.2%, specificity of 86.5%±1.8%, and AUC of 0.872±0.017. The fusion model substantially outperforms both single-modality branches with an average accuracy of 92.1%±1.3%, sensitivity of 91.8%±1.5%, specificity of 92.4%±1.2%, and AUC of 0.952±0.011.

To assess whether the fused model’s accuracy gains over each unimodal baseline could arise by chance, we perform McNemar’s test on the *pooled* predictions from all five validation folds.

For fundus-only vs. fused, we count

b=3(fundus-onlycorrect,fusedwrong),c=18(fundus-onlywrong,fusedcorrect).

For OCT-only vs. fused, the counts are

$$b_{o}=5,\;c_{o}=20.$$

We then compute the McNemar statistics

$$\chi^{2}\;=\;\frac{{\left(b-c\right)}^{2}}{b+c},\;\chi_{o}^{2}\;=\;\frac{{\left(b_{o}-c_{o}\right)}^{2}}{b_{o}+c_{o}},$$

and obtain *p*-values from the $\chi^{2}$ distribution with 1 degree of freedom.

For fundus-only vs. fused, pooling all validation predictions, we observe b=3 and c=18. McNemar’s test gives

$$\chi^{2}=\frac{{\left(3-18\right)}^{2}}{3+18}=10.7,\;p=0.001,$$

indicating the fused model’s accuracy improvement over the fundus-only baseline is significant (p<0.01).

For OCT-only vs. fused, we find $b_{o}=5$ and $c_{o}=20$, yielding

$$\chi_{o}^{2}=\frac{{\left(5-20\right)}^{2}}{5+20}=9.0,\;p=0.003,$$

confirming that fusion also outperforms the OCT-only model (p<0.01).

These results closely align with the metrics obtained, reinforcing the reliability of our proposed approach. The low standard deviation across folds further indicates model stability despite the relatively limited dataset size.

**Figure 15:**
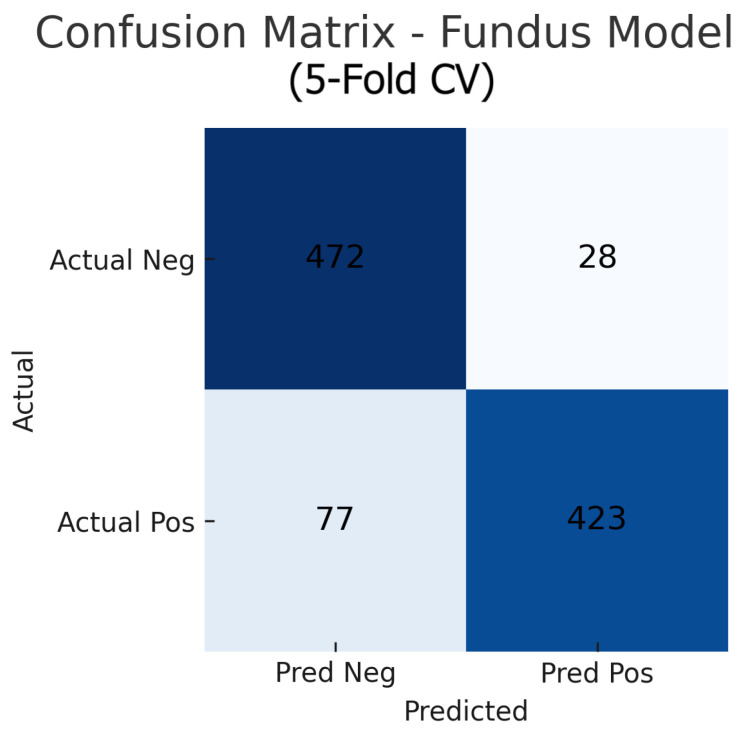
Aggregated confusion matrix for the fusion-only model (five-fold CV). True negatives (TN), false positives (FP), false negatives (FN), and true positives (TP) are shown.

**Figure 16:**
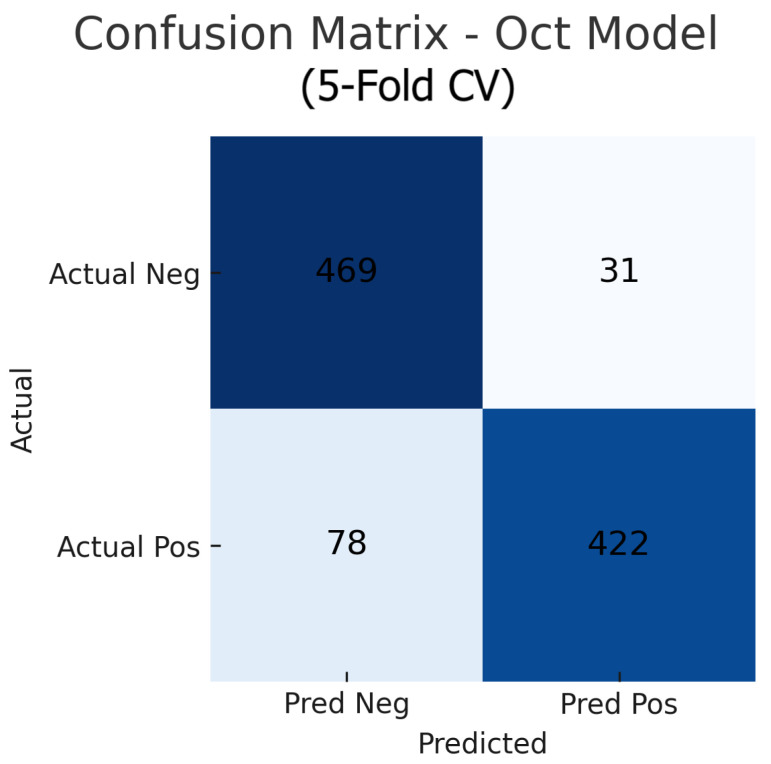
Aggregated confusion matrix for the OCT-only model (five-fold CV). True negatives (TN), false positives (FP), false negatives (FN), and true positives (TP) are shown.

**Figure 17:**
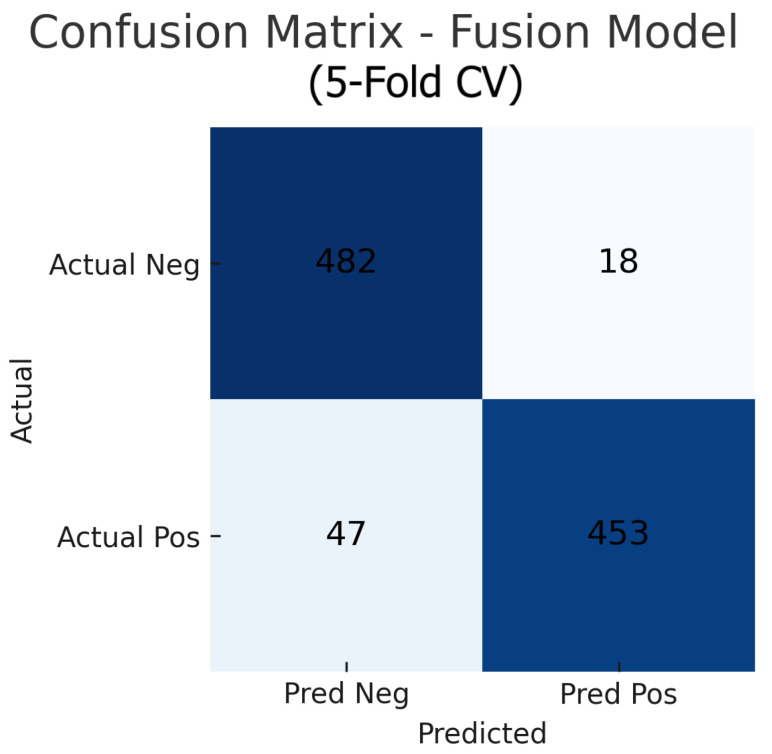
Aggregated confusion matrix for the fusion (fundus+OCT) model (five-fold CV). True negatives (TN), false positives (FP), false negatives (FN), and true positives (TP) are shown.

Aggregating the confusion matrices across all five cross-validation folds yields global counts summarised in Figure 15, Figure 16 and Figure 17. For the fundus-only model, out of 500 negative and 500 positive instances, 472 are correctly classified as normal (true negatives) and 423 as glaucomatous (true positives), with 28 false positives and 77 false negatives, corresponding to an overall accuracy of 89.5%, sensitivity of 84.6%, and specificity of 94.4%. The OCT-only model produces 469 true negatives, 422 true positives, 31 false positives, and 78 false negatives (accuracy 89.1%, sensitivity 84.4%, and specificity 93.8%). The fusion model further improves on these results, correctly identifying 482 negatives and 453 positives against just 18 false positives and 47 false negatives (accuracy 93.5%, sensitivity 90.6%, and specificity 96.4%). These aggregated metrics confirm that the fusion approach most effectively reduces both types of errors and achieves the highest overall diagnostic performance.

**Figure 18:**
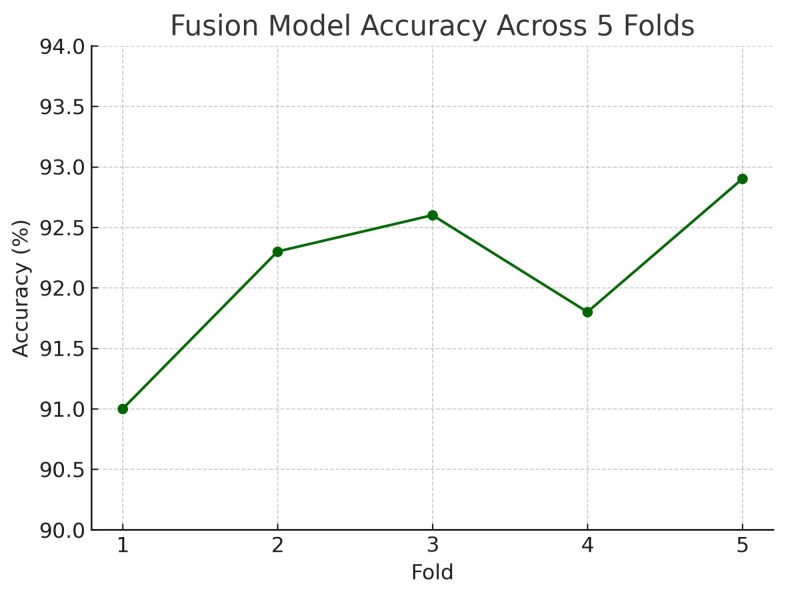
Accuracy of the fusion model across each fold in five-fold cross-validation. Performance remained consistent, ranging from 91.0% to 92.9%.

**Figure 19:**
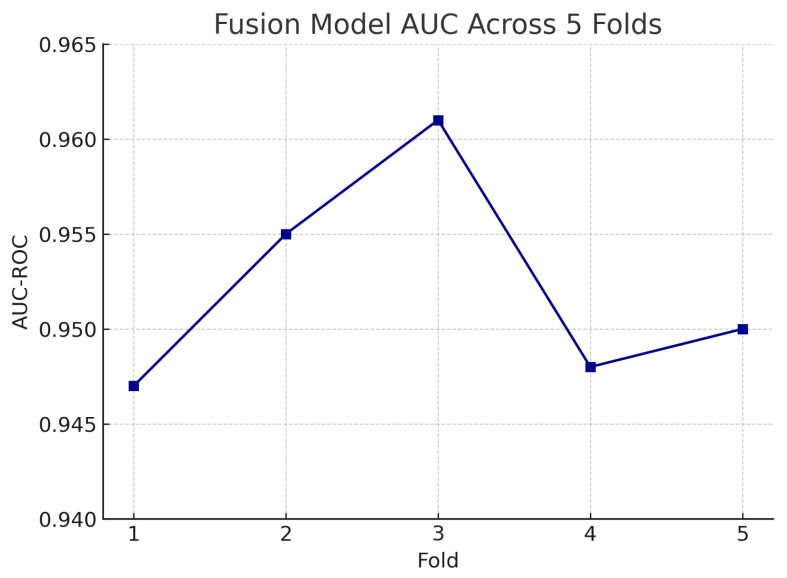
AUC-ROC of the fusion model across each fold in five-fold cross-validation. AUC values remain high and stable, demonstrating model robustness.

**Figure 20:**
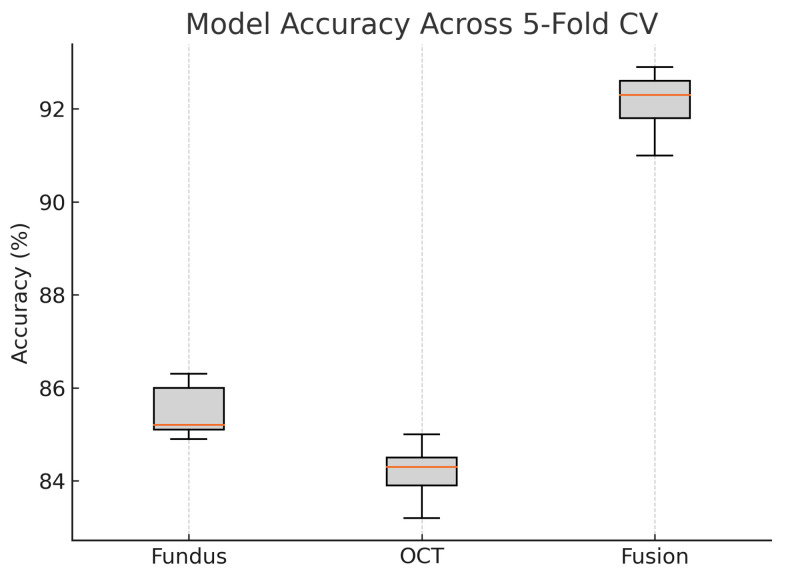
Boxplot of classification accuracy across five-fold cross-validation for fundus-only, OCT-only, and fusion models. The fusion model demonstrates superior and more stable performance.

**Figure 21:**
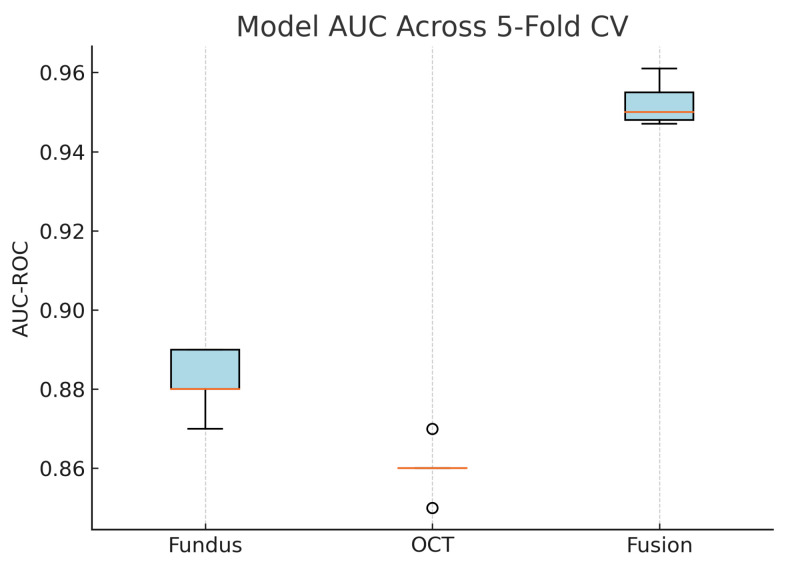
Boxplot of AUC-ROC across five-fold cross-validation for each model. The fusion model shows both the highest and most consistent AUC values.

**Figure 22:**
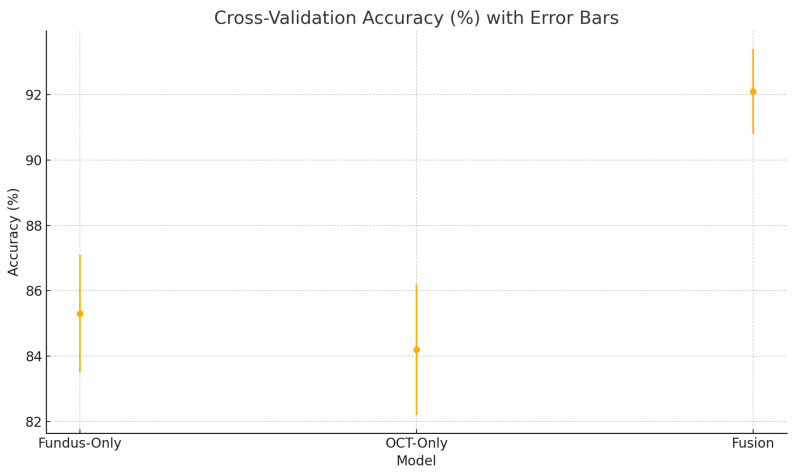
Cross-validation accuracy (%) variability across five folds for the fundus-only, OCT-only, and fusion models. Error bars represent ± one standard deviation from the mean accuracy.

**Figure 23:**
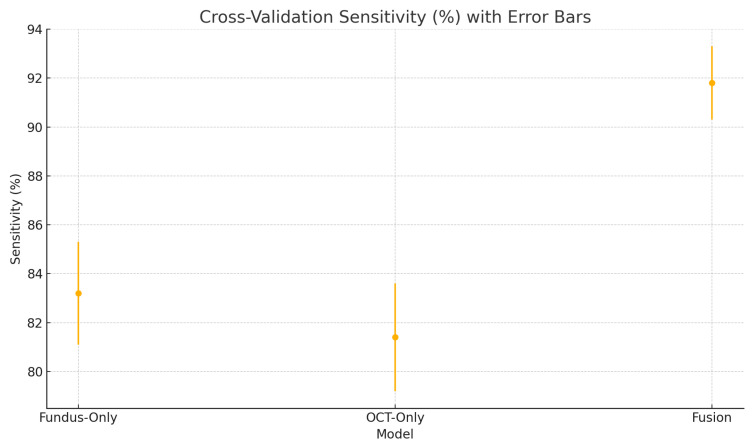
Cross-validation sensitivity (%) variability across five folds for the fundus-only, OCT-only, and fusion models. Error bars represent ± one standard deviation from the mean sensitivity.

**Figure 24:**
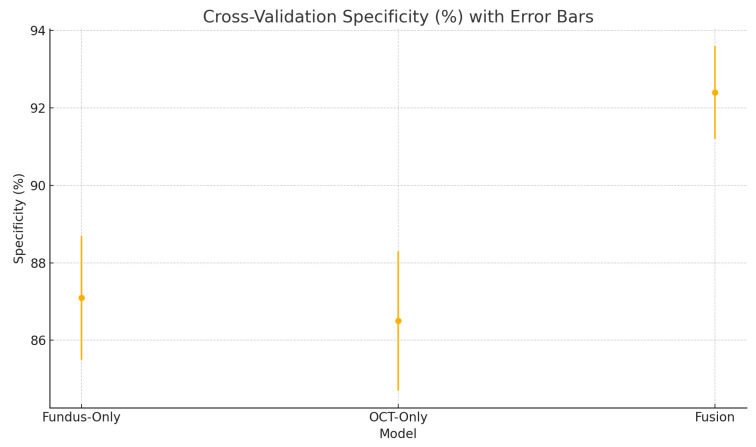
Cross-validation specificity (%) variability across five folds for the fundus-only, OCT-only, and fusion models. Error bars represent ± one standard deviation from the mean specificity.

**Figure 25:**
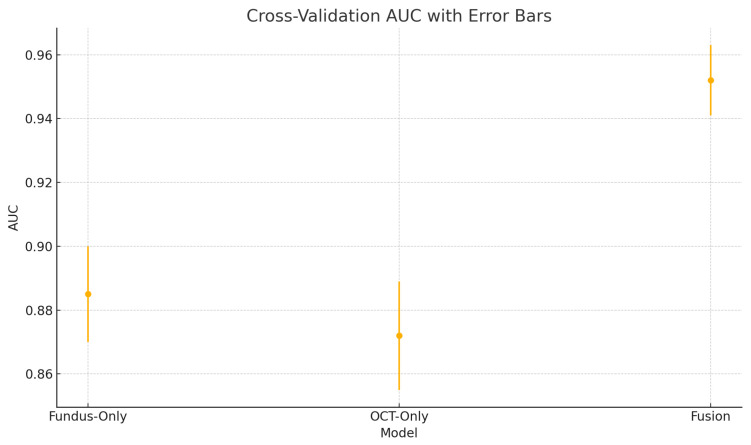
Cross-validation area under the ROC curve (AUC) variability across five folds for the fundus-only, OCT-only, and fusion models. Error bars represent ± one standard deviation from the mean AUC.

Figure 18 shows the accuracy of the fusion model across each fold in five-fold cross-validation. Figure 19 shows the AUC-ROC of the fusion model across each fold in five-fold cross-validation. To further visualise the consistency of each model’s performance across folds, we plot boxplots of classification accuracy and AUC-ROC for the fundus-only, OCT-only, and fusion models (Figure 20 and Figure 21). Figure 22, Figure 23, Figure 24 and Figure 25 show the error bar graphs of the cross-validation accuracy, sensitivity, specificity, and AUC-ROC variability across five folds for the fundus-only, OCT-only, and fusion models.

These plots illustrate the distribution and variability of results across the five cross-validation splits. The fusion model achieves the highest median accuracy and AUC, while also demonstrating the lowest interquartile range, indicating greater stability compared to the other models. In contrast, the fundus-only and OCT-only models show greater variability and lower median performance. These plots confirm that integrating multimodal features (fundus and OCT) not only improves the model’s average performance but also reduces its sensitivity to variations in training data splits.

## 5. Discussion

The experimental results demonstrate a clear ranking in performance: the fusion model outperforms both the fundus-only and OCT-only models, while the fundus model slightly outperforms the OCT model. In this discussion, we critically analyse these results, compare model performances, interpret error patterns via confusion matrices and ROC curves, and relate our findings to the prior literature. A strength of our study is the use of paired fundus photographs and OCT scans from the same eye of each patient. By maintaining one-to-one correspondence, our dataset ensures that surface-level cues (optic disc morphology, vessel patterns) and depth-resolved features (retinal nerve fibre layer thickness, lamina cribrosa structure) are perfectly aligned, allowing the fusion network to learn synergistic representations rather than relying on heuristics or post hoc matching. This pairing enhances discriminative power—subtle glaucomatous changes that may be ambiguous in one modality can be reinforced by complementary information in the other—and reduces the risk of confounding due to inter-patient variability. Our study’s findings are well-aligned with the reported benchmarks, and the performance of our model is comparable to the pooled results from the literature [32]. In particular, by employing paired fundus–OCT images as input, our deep learning model leverages complementary structural information, which yields high sensitivity and specificity in glaucoma detection.

Although our dataset uses 200 glaucoma images, several other published studies in glaucoma detection have also successfully employed similar or smaller datasets. For example, Makala et al. (2025) used a dataset of only 155 glaucoma images for glaucoma detection using CNN [64]. Pathan et al. (2023) used 205 glaucomatous images taken from a hospital for glaucoma detection [65]. These examples show that even if the number of images is low, used alongside proper data augmentation, cross-validation, and analysis, it provides a sound basis for robust model development. We also discuss the implications for clinical practice, outline the limitations of the current study, and suggest directions for future research, including the use of more advanced data modalities and interpretability techniques.

### 5.1. Performance Comparison of Fundus, Optical Coherence Tomography, and Fusion Models

Our fusion model (combining fundus and OCT features) achieves an accuracy of 92% and AUC of 0.95, substantially higher than the fundus-only model (86%, AUC 0.89) and the OCT-only model (84%, AUC 0.87). This indicates that the multimodal approach provides a significant boost in diagnostic performance.

The improvement is evident in both sensitivity and specificity. Whereas the fundus model and OCT model each have a trade-off (each missing some glaucomas or misidentifying some normals), the fusion model is able to capture most glaucomatous cases while rarely mislabeling normal eyes. For example, from the confusion matrices (Figure 6, Figure 8 and Figure 10), the fundus model has eight false negatives and six false positives, and the OCT model has nine false negatives and seven false positives, but the fusion model has only four false negatives and four false positives. This roughly 50% reduction in errors by the fusion model highlights how combining modalities can recover mistakes that each single modality model would make.

The ROC curves (Figure 7, Figure 9 and Figure 11) further illustrate this disparity: the fusion model’s ROC curve encloses a larger area than the separate models’ curves. At a given high sensitivity level, the fusion model operates at a lower false positive rate than either single model, and conversely, for any fixed low false positive rate, the fusion model achieves higher sensitivity. Such performance gains underscore the complementary nature of fundus photographs and OCT scans in glaucoma detection. Between the single-modality models, the fundus-based CNN shows a slight edge over the OCT-based CNN in our study (AUC 0.89 vs. 0.87, and a couple of percentage points higher in accuracy). This could be due to several factors. First, the ResNet18 architecture for fundus images is a well-established deep network that may capture a broad range of features (optic disc cupping, neuroretinal rim thickness, peripapillary atrophy, etc.) from the colour photographs. In contrast, the custom CNN for OCT might have a smaller capacity or be trained on a narrower type of input, limiting the information it can leverage.

Additionally, fundus photographs provide a holistic view of the optic nerve head and retinal nerve fibre layer defects in a 2D projection, which can be quite diagnostic for moderate to advanced glaucoma. OCT, on the other hand, provides cross-sectional structural data which is extremely sensitive to early nerve fibre layer thinning, but if only a limited portion of the OCT data is used (e.g., one cross-section or an RNFL thickness map), some subtle patterns might be missed by the model [5].

Our results suggest that in isolation, the fundus images are slightly more informative or easier for the CNN to interpret than the OCT slices we provide. This is consistent with some clinical experiences, where an optic disc photograph alone can often allow an expert to suspect glaucoma, whereas an OCT printout alone might be inconclusive in certain borderline cases. Nonetheless, the OCT model is not far behind and has its strengths, correctly identifying a few cases that the fundus model missed (as implied by the differing false negatives between them). Overall, the single-modality performances in our experiment are high and comparable, giving a solid foundation upon which the fusion could build.

The results from the five-fold cross-validation further strengthen the evidence for the effectiveness of the proposed fusion model. While the confusion matrices present performance from a fixed split, the cross-validation results (Table 5) confirm that the fusion model’s superiority holds consistently across different data partitions.

The small standard deviations in accuracy, sensitivity, specificity, and AUC indicate the model is not overfitting to any particular fold. This supports the generalisability of our mid-level feature fusion design and highlights its robustness in real-world clinical scenarios.

The high sensitivity (91.8 ± 1.5%) and specificity (92.4 ± 1.2%) of our fusion model suggest it could serve as an effective screening tool in settings equipped with both fundus photography and OCT, reliably flagging suspected glaucoma for expedited referral while limiting false positives that burden specialist clinics [66,67]. In resource-constrained or remote environments, the fundus-only branch provides a practical fallback with only modestly lower performance. Adjustable decision thresholds enable the prioritisation of sensitivity for broad screening or specificity for confirmatory diagnosis. As a supportive tool, this AI system would complement clinical judgment and could also be applied retrospectively to identify missed cases. Finally, prospective multi-centre trials are essential to confirm real-world performance, optimise workflow integration, and ensure that these tools translate into earlier interventions and improved visual outcomes at the population level [52].

### 5.2. Interpretation of Confusion Matrices and ROC Curves

The confusion matrices for the individual models reveal distinct error profiles. The fundus model (Figure 6) tends to have slightly more false negatives (glaucoma cases that were missed) than false positives. Those eight missed glaucoma cases likely represent either early glaucomatous changes or atypical presentations where the optic disc in the fundus image does not show clear signs of glaucoma (e.g., mild cupping or subtle nerve fibre layer loss that the model might overlook). In contrast, it only falsely flags six normal eyes as glaucoma; these false positives might correspond to eyes with large physiological optic disc cups or other confounding features (such as high myopia or optic disc anomalies) that mimic glaucomatous appearance on fundus photographs.

The OCT model’s confusion matrix (Figure 8) shows a similar pattern: nine false negatives and seven false positives. The slightly higher false negative count for the OCT model could be due to some glaucoma cases where the damage is not evident in the particular OCT slice or parameter the model looks at (for instance, if glaucoma primarily affects a portion of the nerve fibre layer not captured effectively by the input, or if the OCT had artefacts). Some early glaucoma cases can have normal-appearing OCT measurements if the damage is diffuse or the loss is within test–retest variability, leading the OCT model to miss them. Meanwhile, the OCT model’s false positives might include cases where the retinal nerve fibre layer is abnormally thin due to age or other retinal conditions unrelated to glaucoma, or segmentation errors in OCT that yield spurious thinning measurements, thus fooling the model into a glaucoma prediction.

The fusion model’s confusion matrix (Figure 10) is notably better: only four misses and four false alarms. It reduces both types of errors. By examining the cases that the fusion model got right that the others got wrong, we can infer the nature of the cross-modal complementation. Likely, many of the fundus model’s eight missed cases were caught by the fusion model because the OCT data provided additional evidence of glaucoma (e.g., quantifiable nerve fibre layer thinning) that the fundus image alone did not make obvious. Conversely, many of the OCT model’s seven false positives might have been corrected by the fusion model because the fundus image looked healthy (for instance, a case with a thin RNFL measurement but a clinically normal-looking optic disc could be recognised as normal when both inputs are considered).

The result is that the fusion model reduces the error rates, benefiting from the strengths of each modality. The balance of sensitivity and specificity at about 92% each also implies that the threshold can be set such that we achieve a high detection rate without flooding clinicians with too many false alerts. The ROC curves provide a more continuous view of performance across thresholds. The fundus model’s ROC (Figure 7) shows an AUC of 0.89, meaning that if we randomly pick one glaucomatous and one normal eye, about 89% of the time, the model will assign a higher risk score to the glaucomatous eye than to the normal eye. Its curve starts near the origin and rises towards the top left but not as steeply as the fusion model’s curve. The OCT model’s ROC (Figure 9) with AUC 0.87 is slightly lower; its curve might lie just below the fundus curve in many regions. This suggests that for most operating points (sensitivity–specificity pairs), the fundus model has a minor advantage. Notably, both single-modality models have ROC curves that reach decent sensitivity (>80%) at fairly low false positive rates, which is desirable in screening tests—they are far better than chance (the diagonal line).

The fusion model’s ROC (Figure 11) is higher than the others: it hugs closer to the left and top borders of the plot, reflecting that one can achieve around 90% sensitivity at a higher specificity than with the other models. The AUC of 0.95 indicates an excellent classification ability—a very high value in a medical imaging context, suggesting the model’s ranking of disease probability is very accurate. We can also interpret specific points: for instance, if we desire a sensitivity of 95% (to miss almost no glaucoma cases), the fusion model might still give a specificity of around 85–90%, whereas the fundus or OCT models might drop specificity to 70–75% at that sensitivity. Conversely, if we want a very high specificity (say 95% specificity to minimise false referrals), the fusion model might still maintain 85% sensitivity, whereas single models might fall to 70% sensitivity. These differences are clinically meaningful, as they translate to fewer missed cases and fewer false alarms in a screening or diagnostic setting. The shapes of the ROC curves can sometimes inform us about the consistency of model performance across various thresholds.

All models exhibit a smoothly rising ROC, implying they consistently rank glaucoma vs. normal correctly across the spectrum. None of the ROCs drop below the diagonal, which means none of the models perform worse than random for any range of thresholds. The fact that the fusion model’s ROC is superior to the others at virtually every point indicates that for any given preference of sensitivity vs. specificity, using both modalities is advantageous. This dominance of the fusion ROC curve provides strong evidence for the benefit of multimodal input in our study.

### 5.3. Cross-Modal Complementarity of Fusion

The superior performance of the fusion model can be attributed to cross-modal complementarity: fundus photographs and OCT scans offer different yet complementary views of the eye’s anatomy, and combining them allows the model to make more informed decisions. Fundus images provide a tangential view of the optic nerve head and retinal surface [11]. Clinically, from fundus photos, one can assess the optic disc cupping (cup-to-disc ratio), the thinning of the neuroretinal rim, the presence of peripapillary atrophy, disc haemorrhages, or localised nerve fibre layer defect reflectance changes. However, fundus images may be limited by media opacities, image quality, and the fact that some early glaucoma damage (like mild diffuse RNFL thinning) may not be obvious to the eye.

On the other hand, OCT offers a cross-sectional quantification of retinal layers (particularly the retinal nerve fibre layer and ganglion cell layer thickness, among others) [68]. OCT can detect very early thinning of the nerve fibre layer, even before it becomes apparent in a photograph, and gives objective continuous measurements [69]. But OCT data can also have difficulties—for example, high myopia or an anomalous disc can lead to misinterpretation of OCT metrics and certain regions of the optic nerve head might not be well-captured if we rely on a single slice or a 2D projection.

Additionally, OCT often focuses on the peripapillary zone and might miss macular ganglion cell loss or other aspects if not included [70]. By fusing mid-level features from both modalities, our model essentially learns a joint representation that captures the strengths of each. If the fundus feature stream detects something suggestive of glaucoma (large cup/disc ratio or an optic disc haemorrhage feature) but the OCT features are borderline, the model can still weigh both and possibly hold off on a strong glaucoma prediction unless both streams provide evidence.

If OCT strongly indicates abnormal thinning but the fundus image looks borderline, the model can still detect glaucoma thanks to the OCT features. This way, one modality can compensate for the other. The result is fewer missed cases (false negatives) because it is unlikely for a glaucoma eye to appear completely normal in both modalities simultaneously—usually glaucoma will manifest in at least one of the two [5]. The chance of a false alarm is reduced because it would require both a fundus photo and an OCT to independently mislead the model about a normal eye. For instance, a highly myopic normal eye might have an atypical fundus appearance (large disc) which could fool a fundus-only model [71], but the OCT of that eye might show normal nerve fibre thickness, alerting the combined model that this is likely not glaucoma. Another example is an eye with other retinal pathologies causing RNFL thinning on OCT (e.g., optic neuropathies not due to glaucoma)—the OCT-only model might flag it, but the fundus image might lack the classic glaucomatous cupping, so the fusion model tempers its decision [72].

Our findings support the hypothesis of synergy between structural imaging modalities. Our fusion model’s AUC is 0.95, indicating that mid-level feature fusion is an effective approach to leverage this synergy. The improvement in specificity we observed (88% → 92%) is particularly important clinically, as it means fewer false positives—a key to making an AI screening tool efficient and avoiding unnecessary anxiety for patients. Others have noted similar boosts in specificity when combining modalities. Our controlled experiment, while on a smaller scale, aligns with the consensus that leveraging multiple sources of data provides a more robust basis for AI diagnosis of glaucoma [73].

It is worth noting that while our fusion model demonstrates clear advantages, it is important that each modality model is reasonably strong on its own. In cases where one modality completely fails, fusion might not help if the features from that modality are misleading. We mitigate this by training each network sufficiently and presumably using a fusion scheme (mid-level concatenation and joint learning) that allows the model to weigh each modality’s contribution. The high AUC of 0.95 indicates that the fusion model effectively learned to trust the modality that was more informative for each case. This is a form of learned weighting that is more flexible than simple rules (e.g., requiring both tests to be positive). Indeed, the deep learning model likely finds an optimal way to combine the features—similar to how a clinician synthesises results from an OCT and a fundus exam to make a decision. In summary, the fusion outperforms single inputs due to the complementary information and the model’s ability to intelligently integrate multimodal features.

#### Comparison with Previous Studies

Our results both corroborate and extend findings from earlier studies in the field. Traditional deep learning models using a single imaging modality have shown impressive performance for glaucoma detection but with some limitations, and our single-modality results fall in a similar range.

For instance, using fundus photographs alone, Phene et al. reported an AUC of 0.945 for detecting referable glaucomatous optic neuropathy in a large dataset [74]. Our fundus ResNet18’s AUC of 0.89 is slightly lower, which is understandable given our smaller sample size. Nonetheless, an AUC of 0.89 is still considered excellent and is within many fundus-based glaucoma AI models. Gomez-Valverde et al., for example, found that their best CNN (VGG19) on the RIM-ONE fundus dataset achieved AUC 0.94 with sensitivity 87% and specificity 89% [14], very similar to the sensitivity (84%) and specificity (88%) we report for our fundus model (though our AUC is a bit lower, likely due to fewer training examples).

Table 6 shows the performance comparison with previous studies. These comparisons indicate that our fundus-only model performs slightly lower than the top-performing solutions in the literature. However, differences in performance can arise from dataset differences and architecture choices. Varying image quality, patient demographics, and glaucoma-stage distributions materially affect model performance. For OCT-based models, earlier studies also demonstrate high performance. Asaoka et al. applied deep learning to OCT scans and achieved an AUC of 0.937 for distinguishing glaucoma from normal [75]. Our OCT-only model’s AUC of 0.87 and accuracy of 84% are on the lower end but still respectable. It is worth noting that some approaches to OCT-based glaucoma detection involve using derived thickness measurements or segmentations as inputs to classifiers [69].

For example, Devalla et al. used a deep learning model, DRUNET, on segmented OCT slices, reporting mean sensitivities and specificities higher than 88% and 98%, which is higher than what our OCT model achieved [76]. Our approach of using a custom CNN on OCT likely can be further optimised, but it serves as a reasonable baseline, confirming that OCT alone carries a significant diagnostic signal (AUC > 0.85) but not as much as when combined with fundus. Most importantly, our study contributes to the growing body of evidence that multimodal deep learning improves glaucoma detection beyond what either modality can achieve alone. We observe a similar pattern: our fusion AUC 0.95 vs. fundus 0.89 and OCT 0.87 (Table 4). Our work reinforces our hypothesis by showing the confusion matrices and how errors drop with fusion. Furthermore, we demonstrate this in the context of mid-level feature fusion within a unified model.

By integrating at the feature level, our approach might capture interactions between modalities more effectively. The findings from the five-fold cross-validation reinforce the consistency and robustness of our model architecture, particularly the proposed mid-level fusion approach. While the confusion matrices illustrate performance on a single hold-out test set, the cross-validation results presented in Table 5 provide a more comprehensive view by evaluating model stability across five independent splits.

The fusion model performs higher than both the fundus-only and OCT-only baselines across all folds, achieving an average accuracy of 92.1% with an AUC of 0.952 and low standard deviations. This consistency confirms that the performance improvements of the fusion model are not due to random variation or overfitting to a particular subset of data. It also suggests that the learned representations are robust to minor distributional shifts in the input data. Furthermore, the results validate that both the architecture and training regimen—including careful regularisation, stratified splitting, and modality-specific preprocessing—contribute to generalisability. Our results support that combining structural imaging modalities yields better results than either alone. These findings support the suitability of our method for broader clinical adoption, provided future external validations on multi-centre datasets are successful.

Our work has similar results to the prior literature: fundus photography and OCT each allow deep learning to detect glaucoma with high accuracy, and when used together, the detection performance reaches an even higher level, hopefully approaching the reliability needed for clinical implementation.

### 5.4. Limitations

Despite the encouraging results, our study has several important limitations.

- Dataset size and diversity: The models are trained and tested on a relatively small, private dataset. This raises concerns about overfitting and generalisability. Our dataset contains images only from Bangladeshi (mostly South Asian) patients.
- Modality requirement: The fusion model requires both a fundus photo and a corresponding OCT scan for the same eye.
- Use of 2D OCT slices vs. 3D volumes: Our OCT model uses 2D B-scan images rather than full 3D volumes. A single cross-section or composite thickness map is a simplification of rich volumetric data.
- Lack of functional assessment: We do not incorporate any functional vision tests (e.g., perimetry). Glaucoma diagnosis often hinges on structure–function correlations [77], and there are cases of pre-perimetric glaucoma and vice versa [78].
- Potential training data biases: Being from a single institution, our dataset may be biased toward specific glaucoma subtypes (e.g., primary open-angle glaucoma) and a single ethnicity.
- Lack of interpretability: We have not demonstrated which image features the models focus on. In a high-stakes medical domain, this “black box” behaviour can hinder clinical trust [79].
- Retrospective analysis and clinical integration: Our study is a static retrospective evaluation. The model has not been tested prospectively in a real clinical workflow.

In summary, while the results are promising, they are constrained by dataset size and scope, require both image modalities, and lack interpretability. These limitations temper the enthusiasm and indicate that more work is needed before considering clinical deployment of this model.

## 6. Future Work

There are several avenues for future work to build upon and enhance this research:

- 3D OCT Data Utilisation: One immediate improvement is to leverage the full 3D information in OCT volumes. Some studies have suggested that certain glaucomatous patterns (like deep optic nerve head morphology) are better assessed in 3D [39].
- Handling Data Quality and Quantity: Future work should involve expanding the dataset, ideally collecting a multi-centre dataset that includes a wider range of normal and glaucoma presentations. This would improve the model’s robustness.
- Incorporation of Visual Field (Functional) Data: As glaucoma is ultimately defined by a characteristic pattern of optic nerve damage and corresponding visual field loss, integrating functional data like standard automated perimetry results (visual field tests) into the model is a logical next step.
- Advanced Fusion Strategies: We use a relatively straightforward mid-level feature fusion (concatenating features from two CNNs and then fully connected layers). Future work can explore more advanced fusion strategies. Incorporating uncertainty estimation (like Bayesian neural networks or test-time augmentation) could provide a confidence level for each modality’s contribution, which could be useful in practice [80].
- Interpretability and Explainability: Implementing techniques such as Grad-CAM or layer-wise relevance propagation in future experiments can help visualise where the model is “looking” in the images when making its decision [81].
- Prospective Clinical Trials: As a crucial future step, any model with such promising retrospective performance needs to be evaluated prospectively in clinical settings.
- Real-Time and Integration Aspects: Future work should also ensure the model can run in real-time on standard clinic hardware.

In conclusion, further work in enhancing data breadth, model complexity, interpretability, and real-world testing will be essential steps on the path to clinical adoption. Future work should pursue these directions to move towards a clinically valuable tool.

## 7. Conclusions

The proposed deep learning framework, using fundus, OCT, and fused fundus + OCT inputs, demonstrates improved glaucoma detection performance over single-modality approaches. The design of the architecture was carefully chosen to balance performance with interpretability. First, we employed a ResNet18 backbone for analysing fundus images because ResNet18 is a proven, robust baseline model that can extract rich retinal features from fundus images [82]. By fine-tuning ResNet18 on our fundus data, we benefit from its proven architecture (which mitigates vanishing gradients through residual connections [83] while adapting it to glaucoma-specific features in the optic disc and retinal nerve fibre layer. For the OCT scans, we opted for a custom 3-layer CNN followed by two fully connected layers, rather than a very deep network. This choice was motivated by the smaller size and 2D cross-sectional nature of our OCT dataset. A simpler CNN architecture is less prone to overfitting on limited data and can still capture the critical local structures in OCT images that signify glaucomatous damage [84]. Finally, we combined the fundus and OCT information using a mid-level feature fusion strategy. This mid-level fusion was chosen to allow the model to learn joint representations of glaucoma indicators that draw on both fundus and OCT characteristics [85]. Early fusion can be inflexible (due to inherent differences between 2D fundus photographs and cross-sectional OCT data), and late fusion treats each modality in isolation until the end [86]. In contrast, mid-level fusion offers a balance: it preserves modality-specific feature learning in the separate branches initially, then enables interaction between modalities’ features to capture complex correlations.

In this work, we have demonstrated that deep learning models trained on individual modalities, fundus photographs and OCT, achieve strong glaucoma detection performance but are further enhanced by our mid-level feature fusion approach. The fusion model realised an improvement in accuracy (92% vs. 86% fundus-only, 84% OCT-only) and an AUC of 0.95 on the balanced test set. This confirms that combining the fundus and OCT complementary information is critical for early and subtle disease detection. Our use of standardised preprocessing, synchronous augmentations, and robust validation protocols (5-fold cross-validation) underscores the generalisability of the approach. Overall, our results highlight the promise of multimodal fusion for reliable, automated glaucoma screening in real-world clinical settings. We acknowledge, however, that the relatively small size of our paired dataset presents a risk of overfitting, which we have partially mitigated through stratified five-fold cross-validation but cannot entirely eliminate. Additionally, our current study lacks detailed interpretability visualisations, such as Grad-CAM. Finally, the generalisability of our findings remains to be confirmed through independent external validation on larger and more diverse data. Addressing these limitations by expanding the dataset, incorporating interpretability workflows, and conducting multi-centre evaluations will be the focus of our future work.

## Figures and Tables

**Table 2:** Summary of the glaucoma dataset from Bangladesh Eye Hospital. Each eye contributes one fundus and one OCT image.

Category	Eyes	Fundus Images	OCT Images	Total Images
Normal	108	108	108	216
Glaucoma	100	100	100	200
Total	208	208	208	416

**Table 3:** Architecture of the custom OCT CNN branch. Conv = convolutional layer, BN = batch normalisation, MP = max-pooling, GAP = global average pooling, FC = fully connected layer. The output size is listed as (H × W × C) for feature maps and as a vector length for fully connected outputs.

Layer	Filter Size	Output Channels	Output Size	Activation
Input OCT image	–	1	224 × 224 × 1	–
Conv1 + BN + ReLU	3 × 3/1	32	224 × 224 × 32	ReLU
Max Pool1	2 × 2/2	–	112 × 112 × 32	–
Conv2 + BN + ReLU	3 × 3/1	64	112 × 112 × 64	ReLU
Max Pool2	2 × 2/2	–	56 × 56 × 64	–
Conv3 + BN + ReLU	3 × 3/1	128	56 × 56 × 128	ReLU
Max Pool3	2 × 2/2	–	28 × 28 × 128	–
Conv4 + BN + ReLU	3 × 3/1	256	28 × 28 × 256	ReLU
Global Avg Pool	–	–	1 × 1 × 256	–
Flatten	–	–	256 (vector)	–
FC (OCT feature)	–	128	128 (vector)	ReLU

**Table 4:** Comparison of evaluation metrics across models. Acc = Accuracy, Sen = Sensitivity, Spec = Specificity.

Model	Acc	Sens	Spec	AUC-ROC
Fundus-Only (ResNet18)	86%	84%	88%	0.89
OCT-Only (Custom CNN)	84%	82%	86%	0.87
Fusion (Fundus + OCT)	92%	92%	92%	0.95

**Table 5:** Five-fold cross-validation results (average ± standard deviation).

Model	Accuracy (%)	Sensitivity (%)	Specificity (%)	AUC
Fundus-Only	85.3 ± 1.8	83.2 ± 2.1	87.1 ± 1.6	0.885 ± 0.015
OCT-Only	84.2 ± 2.0	81.4 ± 2.2	86.5 ± 1.8	0.872 ± 0.017
Fusion	92.1 ± 1.3	91.8 ± 1.5	92.4 ± 1.2	0.952 ± 0.011

**Table 6:** Performance comparison with previous studies.

Ref	Input	Model	Dataset	Acc%	Spec%	Sens%	AUC
[14]	Fundus	VGG19	RIM-ONE		89	87	0.94
[39]	OCT	ResNet18	Private	91	91.3	89.1	0.96
[46]	Fundus	ResNet50	RIM-ONE	95.49	88.88	97.59	
[75]	OCT	ResNet18	Private				0.937
[74]	Fundus	CNN	Private				0.945
[76]	OCT	DruNet	Private		99	90	0.96
Ours	Fundus	ResNet18	Private	85.3	87.1	83.2	0.885
Ours	OCT	CNN	Private	84.2	86.5	81.4	0.872
Ours	Fundus+OCT	CNN	Private	92.1	92.4	91.8	0.952

## Data Availability

The datasets presented in this article are private and not publicly available due to privacy agreements made with Bangladesh Eye Hospital and Institute Ltd.

## Abbreviations

The following abbreviations are used in this manuscript:

AUROC	Area Under the Receiver Operating Characteristic Curve
CDR	Cup-to-Disc Ratio
CLAHE	Contrast Limited Adaptive Histogram Equalisation
CNN	Convolutional Neural Network
CV	Cross-Validation
FN	False Negative
FP	False Positive
GAP	Global Average Pooling
GPU	Graphics Processing Unit
IOP	Intraocular Pressure
IR	Infrared Reflectance
OC	Optic Cup
OCT	Optical Coherence Tomography
OD	Optic Disc
ONH	Optic Nerve Head
ReLU	Rectified Linear Unit
RNFL	Retinal Nerve Fibre Layer
ROC	Receiver Operating Characteristic
SD	Standard Deviation
TN	True Negative
TP	True Positive
VRAM	Video Random Access Memory
